# Decision‐making under flood predictions: A risk perception study of coastal real estate

**DOI:** 10.1111/risa.17706

**Published:** 2025-01-18

**Authors:** Avidesh Seenath, Scott Mark Romeo Mahadeo, Matthew Blackett

**Affiliations:** ^1^ Environmental Change Institute, School of Geography and the Environment University of Oxford Oxford UK; ^2^ Portsmouth Business School University of Portsmouth Portsmouth UK; ^3^ School of the Environment Coventry University Coventry UK

**Keywords:** coastal flood modeling, flood prediction, real estate risk, uncertainty, willingness‐to‐pay

## Abstract

Flood models, while representing our best knowledge of a natural phenomenon, are continually evolving. Their predictions, albeit undeniably important for flood risk management, contain considerable uncertainties related to model structure, parameterization, and input data. With multiple sources of flood predictions becoming increasingly available through online flood maps, the uncertainties in these predictions present considerable risks related to property devaluation. Such risks stem from real estate decisions, measured by location preferences and willingness‐to‐pay to buy and rent properties, based on access to various sources of flood predictions. Here, we evaluate the influence of coastal flood predictions on real estate decision‐making in the United Kingdom by adopting an interdisciplinary approach, involving flood modeling, novel experimental willingness‐to‐pay real estate surveys of UK residents in response to flood predictions, statistical modeling, and geospatial analysis. Our main findings show that access to multiple sources of flood predictions dominates real estate decisions relative to preferences for location aesthetics, reflecting a shift in demand toward risk averse locations. We also find that people do not consider flood prediction uncertainty in their real estate decisions, possibly due to an inability to perceive such uncertainty. These results are robust under a repeated experimental survey using an open access long‐term flood risk map. We, therefore, recommend getting flood models “right” but recognize that this is a contentious issue because it implies having an error‐free model, which is practically impossible. Hence, to reduce real estate risks, we advocate for a greater emphasis on effectively communicating flood model predictions and their uncertainties to non‐experts.

## 1 INTRODUCTION

**FIGURE 1:**
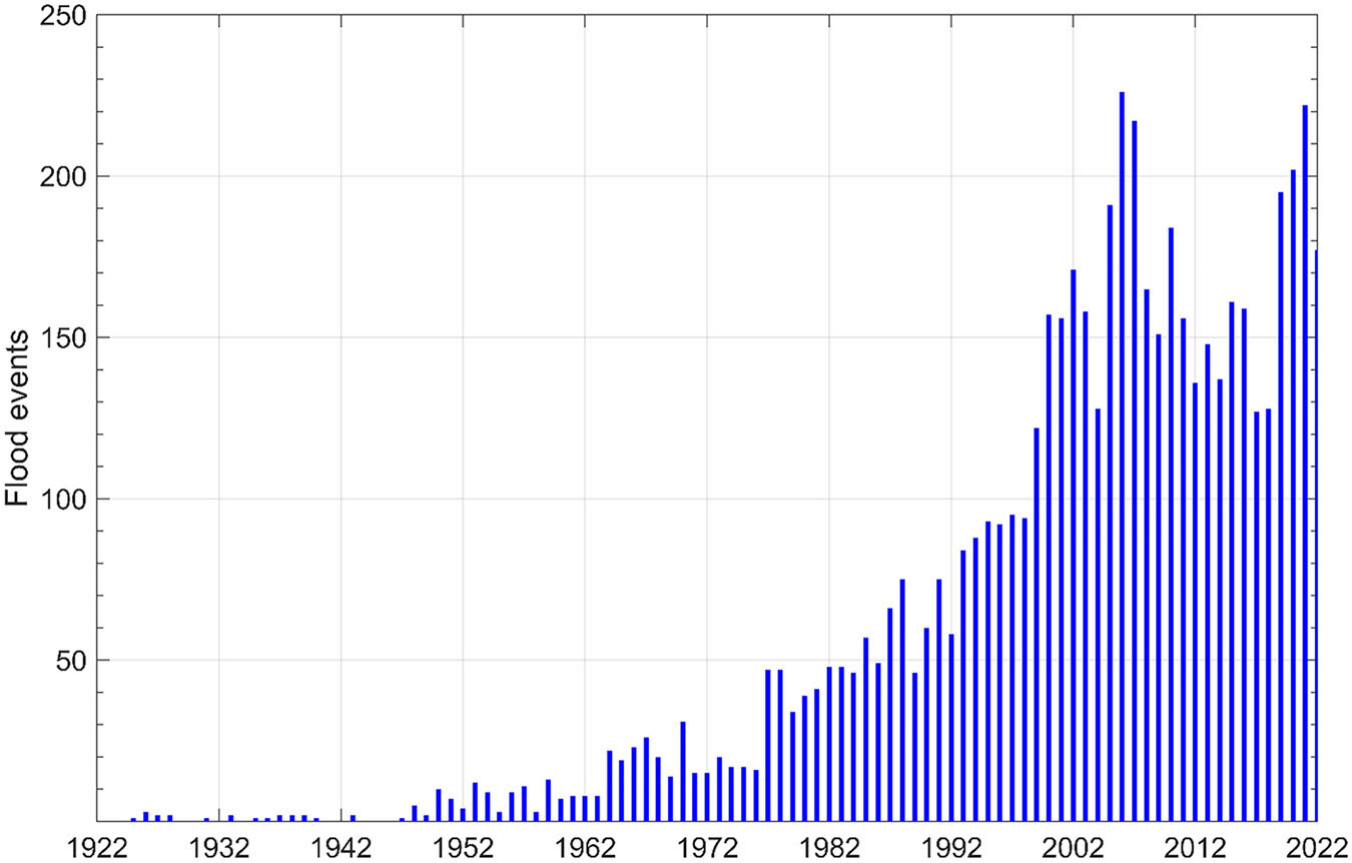
Frequency of flood events recorded globally from 1922 to 2022 (CRED, 2022b).

Flooding is becoming increasingly common globally (Figure 1), and its intensity is likely to increase under future climate projections, with significant socioeconomic impacts (Fan & Davlasheridze, 2016; Lai et al., 2020; Laino & Iglesias, 2023; Park et al., 2023; Rohde, 2023; Tonn & Czajkowski, 2022; Wübbelmann et al., 2023). From 1900 to 2020, flooding has been responsible for ∼7 million deaths and over USD 700 billion in losses globally (Lai et al., 2020). In 2022 alone, flooding in Australia, Bangladesh, Brazil, China, India, Nigeria, Pakistan, South Africa, and the United States collectively affected 43.5 million people, caused USD 135 billion in economic damages, and killed 5539 people (Centre for Research on the Epidemiology of Disasters [CRED], 2022a, 2022b). These socioeconomic impacts are expected to worsen, particularly in low‐lying coastal zones with an elevation of <10 m above mean sea‐level (Kirezci et al., 2023; Moon et al., 2019; Scussolini et al., 2017). These zones—which are often esthetically attractive and economically important (Bin et al., 2008)—are home to over 500 million people who are currently at risk of episodic coastal flooding from storm surges and wave action (Kirezci et al., 2020; Reimann et al., 2023). Recently, between 340 and 630 million people are forecasted to live on land below projected annual flood levels by mid‐century and 2100, respectively (Kulp & Strauss, 2019). Other recent estimates indicate that one billion people live on land less than 10 m above current high tide lines (Kulp & Strauss, 2019). With increases in the rates of sea‐level rise—a significant driver of beach erosion (Leatherman, 2018)—anticipated under future climate projections, the number of people exposed to coastal flooding will inevitably increase. As a quarter of residences within 150 m of the shoreline may be affected by property losses due to beach erosion over the next four decades, the economic effects of erosion‐induced shoreline change are becoming increasingly concerning to beachfront property owners (see Jin et al., 2015, and references therein). Furthermore, coastal flooding is projected to displace 1.46% of the world's population by 2200, where the losses in real global output with and without dynamic economic adaptation of investment and migration are estimated at 0.11% and 4.5%, respectively, underscoring the importance of mitigation strategies (Desmet et al., 2021). In the context of the United Kingdom, the average annual damage to business premises from coastal flooding alone exceeds USD 150 million (Climate Change Committee [CCC], 2021). Hence, flood risk management requires urgent and careful consideration at both national and global levels.

**FIGURE 2:**
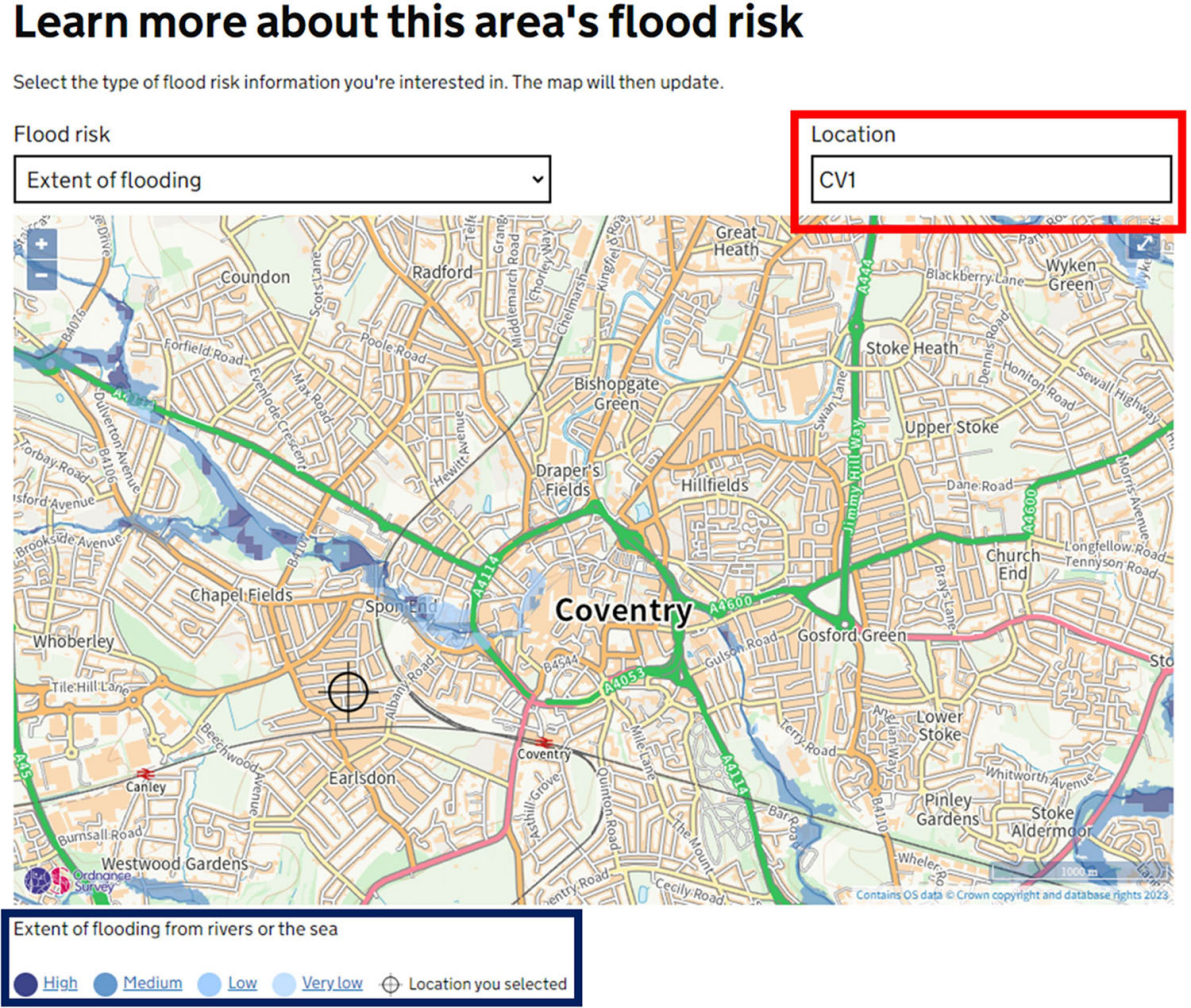
Flood risk predictions from the *Risk of Flooding from Rivers and Seas* (RoFRS) model for the CV1 postcode in the United Kingdom. Red polygon is the postcode. Blue polygon is the flood prediction and associated uncertainty. *Credits*: GOV.UK.

Over the last few decades, behavior‐oriented and physics‐driven flood models have been developed and applied to inform flood risk management (Jodhani et al., 2023; Teng et al., 2017). The former is based on observations rather than the physics behind the observations. The most commonly applied behavior‐oriented flood model is the bathtub model (BTM) , which treats flooding as a function of topography only (areasatriskofflooding=landelevation<floodwaterlevel) (Croteau et al., 2023). This simple functional form makes BTM computationally efficient and easy to apply over large spatio‐temporal scales (Gold et al., 2022; Lopes et al., 2022). In the United Kingdom, BTM principles underpin the Environment Agency's (EA) *Risk of Flooding from Rivers and Seas* (RoFRS) model, which provides long‐term flood risk predictions for areas across England (EA, 2023) (Figure 2). This information is openly available at the postcode level, allowing real estate consumers to get a quick estimate of a property's location flood risk. Physics‐driven flood models, however, are based on the shallow‐water equations derived from depth‐integrating the Navier‐Stokes equations (Bates & De Roo, 2000; Jodhani et al., 2023; Labadie, 1994). These models range in complexity from simulating flow in 1D (channeling flow in cross‐sections) to 2D (using a gridded mesh to simulate flow from one grid cell to the next through a simplification of the shallow water equations) and 3D (using a 3D mesh to simulate flow in x,y,z based on complex fluid equations) (Bates & De Roo, 2000; Jodhani et al., 2023; Labadie, 1994). As flood models increase in complexity from 1D to 3D, greater parameterization is needed, which may be unnecessary for flood simulations (Teng et al., 2017; Zhang et al., 2020). This means that 3D models are more computationally demanding and account for more local environmental factors in flood simulations than 2D and 1D models, which apply more simplifying assumptions. 2D models and coupled 1D/2D models, which often provide a good compromise between complexity and computational efficiency, are more commonly used to inform flood risk management because they tend to facilitate more robust simulations over multi‐storm events across several kilometers (J. T. Samarasinghe et al., 2022; Teng et al., 2017). However, these models, like all flood models, are inherently uncertain, which can compromise flood risk management decisions.

British statistician George Box famously remarked *all models are wrong, but some are useful*. Although this quote was in reference to statistical models, it is equally applicable to flood models, which fail to represent all complexities of flood physics (by applying simplifying assumptions) but can still be useful. Despite this, these models, which are still evolving, represent our best knowledge of flood events. The simplifying assumptions, boundary conditions data, and parameterization that underpin the application of flood models contain inherent and sometimes unavoidable errors, which cause flood predictions to be uncertain (Bales & Wagner, 2009; Bates, 2023; Teng et al., 2017). For example: (a) boundary conditions data inherently contain errors linked to its acquisition and resolution; and (b) the parameterization of models often requires the specification of constants (e.g., bed friction), which may not be characteristic of spatio‐temporal variations in local factors. Although there are no error‐free flood models, we do know which model structures produce good results based on extensive model validation studies in the last two decades (Aronica et al., 2002; Horritt & Bates, 2001; Neal et al., 2012; Seenath et al., 2016; Shustikova et al., 2019; Smith et al., 2011; Willis et al., 2019). Yet, even these “good model structures” are limited to specific types of terrain (Bates, 2023; Seenath, 2018). Therefore, caution is needed when using flood predictions to inform flood risk management, as these predictions are increasingly sought after by the banking, insurance, and real estate sectors, with implications for the economy and society (Bates, 2023; Seenath et al., 2016). For example, flood model overpredictions can: (a) force people to pay higher flood insurance premiums than are necessary, as flood predictions are a common input into insurance costing (Borsky & Hennighausen, 2022; Lea & Pralle, 2021); (b) cause property devaluation in areas erroneously classified as flood vulnerable (Gourevitch et al., 2023; Pryce & Chen, 2011), which adversely impacts wealth (Cronin & McQuinn, 2023); and (c) lead to lost economic opportunities and forced migration (Seenath et al., 2016).

There is considerable awareness of the uncertainty in flood predictions and associated challenges within the flood modeling community (Aronica et al., 2002; Aronica et al., 1998; Bales & Wagner, 2009; Bates, 2023; Teng et al., 2017; Willis et al., 2019). Hence, recent flood modeling studies have adopted probabilistic modeling approaches (Wei et al., 2023; Yulianto et al., 2023; Ziya & Safaie, 2023), which account for the effects of intrinsic uncertainty in models (Domeneghetti et al., 2013; Thompson & Frazier, 2014). Such approaches also enable an investigation into potential outcomes that may occur due to natural variability in stochastic forcing conditions and provide a probabilistic distribution of flood hazard events (Domeneghetti et al., 2013; Thompson & Frazier, 2014). Probabilistic modeling, therefore, makes end users aware of the uncertainties in flood predictions and the likely implications that may arise from flood management decisions informed from these predictions. The UK RoFRS model is also a good example of a probabilistic flood model, as it indicates areas at high, medium, low, and very low chance of flooding per year (Figure 2). Although probabilistic flood models are useful for informing more robust flood risk management decisions, there are still considerable perception risks with making flood predictions from these models openly accessible (Rajapaksa et al., 2016; O. Samarasinghe & Sharp, 2010). This risk relates to property devaluation, which stems from how much people are willing‐to‐pay to buy and rent properties based on flood predictions and is likely to be dependent on their ability to perceive the uncertainty in flood predictions, their level of risk aversion, and their flood experiences and awareness, and whether they are interested in buying or renting a property.

Within the aforementioned context, real estate studies have shown that knowledge and experience of flooding tend to have adverse effects on the real estate market. For instance, properties affected by flooding attract a negative premium in the immediate short‐term (days to a decade) after an event but tend to revert to pre‐flood values with time (Atreya & Ferreira, 2015; Beltrán et al., 2019; Bin & Landry, 2013; Bin & Polasky, 2004; Morgan, 2020; Pommeranz & Steininger, 2020). This temporal variation in real estate market behavior around flood events can have a lasting subconscious effect on flood victims’ real estate decision‐making in response to flood risk information (such as flood maps) (Kellens et al., 2013; Pilla et al., 2019). For example, they might view such information through a binary lens rather than through a probability lens. This implies that flood victims may assume a property will actually flood in the now (present day) if it is located in a flood prediction zone rather than perceiving the property to be at “risk” of flooding, where risk refers to the chance that the property may be exposed to flooding in a particular future scenario. Flood victims may also perceive more dangerous, larger flood likelihood (and consequences), and less personal control than others (Lin et al., 2007).

Furthermore, people interested in buying and renting a property may respond differently to flood risk maps, based on divergent perspectives on long‐term investment versus short‐term occupancy risks. Buying a property is a long‐term investment, and, hence, sale prices may reflect long‐term perceptions of a property value and its associated risk of hazards (Hennighausen & Suter, 2020). Properties in areas perceived to be at higher flood risks are, therefore, likely to experience lower real estate demand. Conversely, renters’ real estate decisions tend to be driven by affordability and convenience (Buchanan et al., 2019). Therefore, in the rental real estate market, there may be greater demand for properties in locations that are predicted to have a higher risk of coastal flooding, as such properties may be perceived to have: (a) lower rental values, and (b) easier access to amenities because of the high social, economic, and cultural values attached to coastal zones.

In the United Kingdom, potential property purchasers are usually required to run conveyancing searches as part of the process of obtaining a mortgage, and these searches provide assessments of flood risk, often informed from various flood models and data that are not publicly available. As various sources of flood model predictions are becoming more accessible through online flood maps, their real estate implications must be understood. Although such maps are undeniably important for flood risk management, there may be ripple effects for the economy and society as the “lay person” accesses the data, and these need careful consideration. Such knowledge, currently unknown, is essential for refining the development and application of flood models. We, therefore, aim to *evaluate the influence of access to multiple sources of flood model predictions on real estate decision‐making, measured through willingness‐to‐pay (WTP)* *for properties and location preferences*, with specific focus on the residential coastal real estate market in the United Kingdom. We do this through an interdisciplinary approach, involving flood modeling, novel experimental WTP real estate surveys of 731 United Kingdom residents (532 from our main survey instrument and 199 from our robustness experiment survey), statistical modeling, and geospatial analysis. We make the distinction between the sale and rental real estate markets in the conceptualization of the study to consider plausible assumptions that prospective homeowners are more concerned than renters with certain factors, such as the proximity to amenities (see, e.g., Pilla et al., 2019) or the risk of damage to assets from flooding (see, e.g., Buchanan et al., 2019), all of which can ultimately influence how our survey participants make location preferences and WTP decisions. The following sections outline our case study location, methods, results, and wider implications of our findings.

## 2 CASE STUDY SITE

**FIGURE 3:**
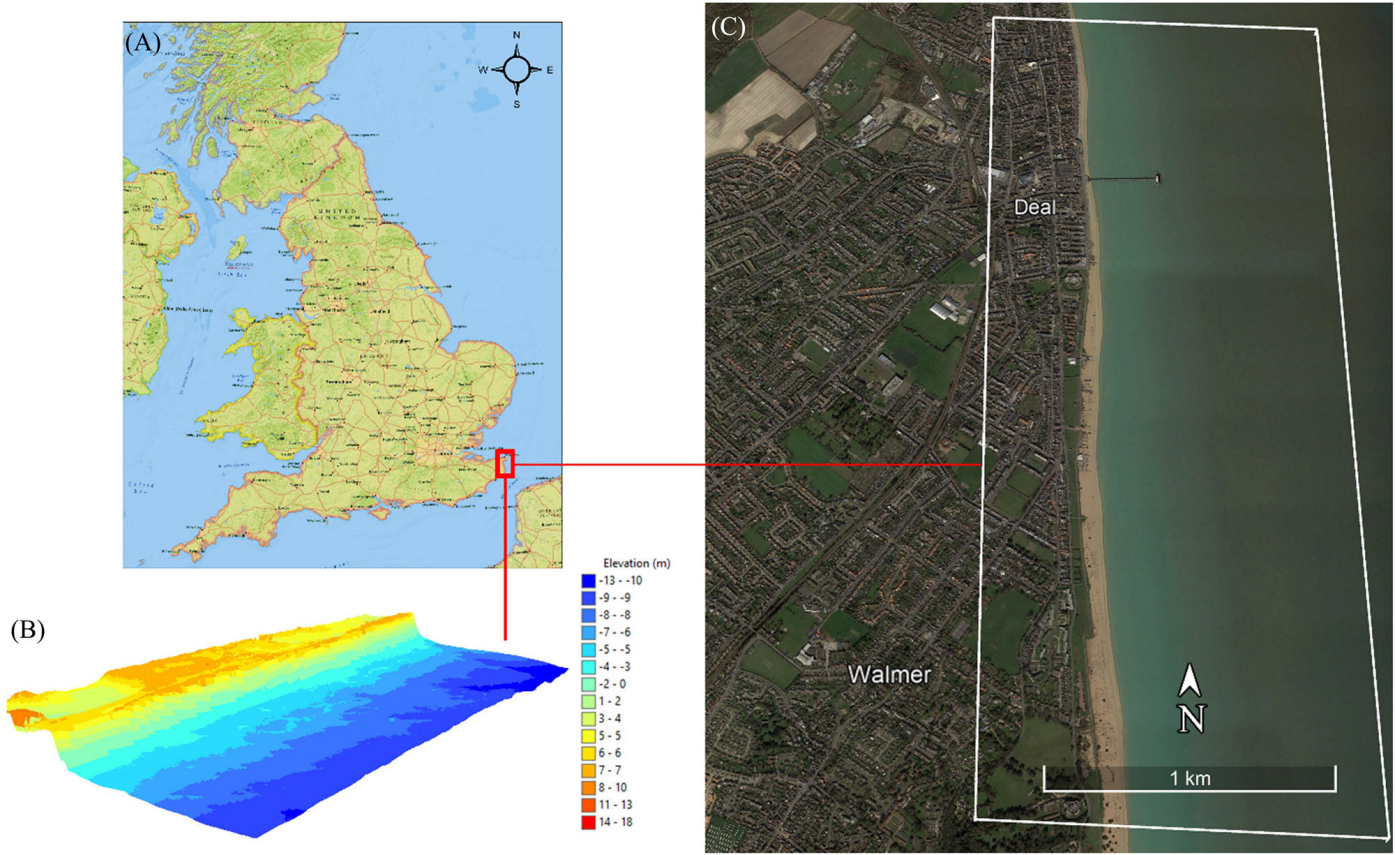
Case study site. (A) Location in the United Kingdom. (B) 3D planimetric view of the site topo‐bathymetry. (C) Satellite view of the site features. White box in (C) outlines the spatial extent of our study site. *Credits*: ESRI National Geographic World Basemap (A), UK Environment Agency 2019 SurfZone digital elevation models (DEM) (B), and Google Earth (C).

Our case study site is a ∼1.6 km^2^ coastal town in Deal, extending ∼0.5 km from land to sea and ∼3.45 km along Sandwich Bay on the east coast of Kent, United Kingdom (Figure 3). This location is a quintessential British coastal town, fronted by over two miles of mixed sand and shingle beaches, with access to a commercial high street, wide paved boardwalks, and all amenities, including shops, health, emergency, protective, hospitality and childcare services, schools, and so forth. The town is also located in relatively close proximity to a university and is well‐served by public transportation with easy commute links to two international airports. Here, for a two‐bedroom house, the average selling price is £275,000, and the monthly rental cost is £975 (Office for National Statistics [ONS], 2023). The area is relatively flat with a straight shoreline, mainly managed by sediment redistribution. The nearshore has a steep upper beach and gentler lower beach (Figure 3).

We select this site because it is in a data‐rich location, with high‐resolution digital elevation models (DEMs) and tide data, to facilitate the flood modeling campaign of our study. As we aim to evaluate the influence of access to multiple sources of flood model predictions on coastal real estate decisions in the United Kingdom, any coastal town with adequate data is suitable for our study. We emphasize that our study adopts an experimental approach and is *not* designed to undertake physically realistic coastal flood vulnerability assessments.

## 3 METHODS AND DATA

### 3.1 Flood modeling

We consider four flood models: three applied in LISFLOOD−FP ranging in complexity and representative of the range of physics‐driven models that are typically applied to inform flood risk communications and management, and BTM applied through ArcGIS10.8.1. The application of all four models in our study enables us to quantify whether uncertainty in flood model predictions influences coastal real estate decisions. Residents in England, for example, can access multiple sources of flood prediction information, which are openly available and informed from computationally different models. These include: (a) a national long‐term flood risk map informed by the RoFRS model, which is built on BTM principles (Section 3.1.2) and provides flood risk information at postcode level (EA, 2023); (b) city council flood maps often informed by physics‐driven models, characteristic of the numerical flow solvers within LISFLOOD−FP; (c) flood risk reports during the conveyancing process of purchasing a new home, which are compiled using information from various flood data sources (e.g., British Geological Society, Land Registry) and consultancy‐based flood models. Sources of uncertainty in flood models include their computational form, setup, and input data. An inability to perceive the uncertainty in flood predictions, evident from conflicting flood prediction sources, may likely result in considerable uncertainty in real estate demand decisions, with non‐trivial implications for a wide range of stakeholders—real estate agents, insurance companies, banks, policymakers, and the public (see, e.g., Rajapaksa et al., 2016). Hence, we need to understand whether there are potential real estate risks associated with access to conflicting sources of such predictions, as a first step toward refining the application of flood models for both managing and communicating flood risk.

#### 3.1.1 LISFLOOD‐FP

LISFLOOD−FP is a well‐documented 2D hydrodynamic model, based on a structured‐grid raster DEM. It predicts water depths in each cell of the DEM at each time‐step in a simulation based on hydraulic continuity principles (Bates et al., 2005). LISFLOOD−FP contains several numerical solvers to simulate flood wave propagation based on some form of the following 2D shallow‐water equations (Sharifian et al., 2023):

(1) $$\frac{\partial h}{\partial t}+\frac{\partial q_{x}}{\partial x}+\frac{\partial q_{y}}{\partial y}=0$$

(2) $$\begin{array}{cc} \underset{\mathrm{acceleration}}{\underset{︸}{\frac{\partial q_{x}}{\;\;\;\;\partial t\;\;\;\;}}}+ & \underset{\mathrm{pressure}}{\underset{︸}{\frac{\partial \left(gh^{2}/2\right)}{\partial x}}}+\underset{\mathrm{advection}}{\underset{︸}{\frac{\partial \left(q_{x}^{2}/h\right)}{\partial x}+\frac{\partial \left(q_{x}q_{y}/h\right)}{\partial y}}}\\ & +\underset{\mathrm{bed}\;\mathrm{gradient}}{\underset{︸}{gh\frac{\partial z}{\partial x}}}+\underset{\mathrm{friction}}{\underset{︸}{\frac{gn_{M}^{2}\left|q_{x}\right.\left|q_{x}\right.}{h^{7/3}}}}=0 \end{array}$$

(3) $$\begin{array}{cc} \underset{\mathrm{acceleration}}{\underset{︸}{\frac{\partial q_{y}}{\;\;\;\;\partial t\;\;\;\;}}}+ & \underset{\mathrm{pressure}}{\underset{︸}{\frac{\partial \left(gh^{2}/2\right)}{\partial y}}}+\underset{\mathrm{advection}}{\underset{︸}{\frac{\partial \left(q_{y}^{2}/h\right)}{\partial y}+\frac{\partial \left(q_{x}q_{y}/h\right)}{\partial x}}}\\ & +\underset{\mathrm{bed}\;\mathrm{gradient}}{\underset{︸}{gh\frac{\partial z}{\partial y}}}+\underset{\mathrm{friction}}{\underset{︸}{\frac{gn_{M}^{2}\left|q_{y}\right.\left|q_{y}\right.}{h^{7/3}}}}=0 \end{array}$$

where Equation (1) is the mass conservation, and Equations (2) and (3) is the momentum conservation equations in x and y Cartesian direction, respectively, h is water depth, t is time, g is gravity, z is bed elevation, $n_{M}$ is Manning's friction coefficient, $q_{x}$ is volumetric flow rate in x direction, and $q_{y}$ is volumetric flow rate in y direction.

LISFLOOD−FP has been extensively developed and validated following its release, becoming a state‐of‐the‐art flood model for application across local to continental spatial scales (Bates et al., 2005; Neal et al., 2018, 2011; Rahimzadeh et al., 2019; Sadeghi et al., 2022; Sharifian et al., 2023; Shaw et al., 2021; Shustikova et al., 2020). It has been successfully applied in fluvial (O'Loughlin et al., 2020; Sanyal et al., 2013; Trigg et al., 2009), coastal (Bates et al., 2005; Seenath, 2018; Wadey et al., 2013), and urban (Chen et al., 2018; Sampson et al., 2012; Sun et al., 2022) environments, with a proven ability to provide results equivalent to and, in some cases, more accurate and reliable than those from more complex 2D flood models (e.g., TELEMAC−2D) at a computationally effective cost (Horritt & Bates, 2001; Seenath et al., 2016; Shustikova et al., 2019). For this reason, we consider LISFLOOD−FP alongside the fact that its results are easily integrated into Geographic Information Systems for flood mapping (Seenath, 2015). Specifically, we focus on three of its numerical flow solvers:

- LISFLOOD−ROE, which applies Villanueva and Wright (2006) approach to solve all terms in the 2D shallow‐water equations. It is, therefore, computationally demanding and represents the most complex type of flood model that is used to inform flood risk maps and management. As it is computationally demanding, it has not been extensively applied and validated. However, a few studies have shown that LISFLOOD−ROE is capable of producing reliable flood depth predictions relative to other numerical flood models (Neal et al., 2012; Sadeghi et al., 2022; Willis et al., 2019).
- LISFLOOD−ACC, which applies a simplified form of the shallow‐water equations by assuming that the advection term is negligible. It treats flooding as a function of friction, water slopes, and local acceleration. These simplifying assumptions enable a quick simulation of flood flows, making LISFLOOD−ACC particularly advantageous for real‐time flood forecasting. LISFLOOD−ACC is also the most popular flow solver in LISFLOOD−FP and has been subject to extensive model validation studies, with its performance often shown to be equivalent to more complex flood modeling approaches (Le Gal et al., 2023; Neal et al., 2012; Seenath et al., 2016).
- LISFLOOD−FL, which is the least complex flow solver in LISFLOOD−FP, is a zero‐inertia model as it ignores the acceleration and advection terms in the shallow water equations. It treats flooding as a function of friction and water slopes only. Its simple functional form, while appropriate for various flood problems, has been shown to underestimate flood propagation speeds (Bates et al., 2010). We consider it here because it is representative of the reduced‐complexity flood models that have been favored historically by flood modelers and managers (Costabile et al., 2020).

#### 3.1.2 Bathtub model (BTM)

BTM treats flooding as a function of topography, meaning that an area is considered to be flood vulnerable if it is lower in elevation than that of the maximum flood water level being simulated (Seenath et al., 2016). It, therefore, ignores hydraulic connectivity and flood routing physics, often overpredicting flood inundation (Leijnse et al., 2021; Seenath et al., 2016; Williams & Lück‐Vogel, 2020). However, an advantage of BTM over physics‐driven models is its DEM‐only requirement and simple raster calculation process (DEM<maximumfloodwaterlevel), which make it computationally efficient and particularly useful for macroscale (local to continental scales; daily to centennial timescales) applications. For these reasons, BTM commonly underpins flood risk assessment and management globally, particularly in data‐poor regions (Garcia & Dias, 2023; Lopes et al., 2022), despite the considerable awareness of its limitations (Gold et al., 2022; Lopes et al., 2022). The UK RoFRS model is also built on the principles of BTM (EA, 2023), hence its consideration here. Following Seenath et al. (2016), we apply BTM using ArcGIS10.8.1.

#### 3.1.3 Flood scenarios, model setup, and simulations

Our study adopts an experimental approach to investigate location preferences and how much people are willing‐to‐pay to buy and rent coastal properties under two flood scenarios: (a) current flood vulnerability in response to tidal surges, and (b) future flood vulnerability in response to a 1 m sea‐level rise. We use LISFLOOD−ROE, LISFLOOD−ACC, LISFLOOD−FL, and BTM to simulate each flood scenario. We, therefore, run a total of eight flood simulations based on the specifications below.

**FIGURE 4:**
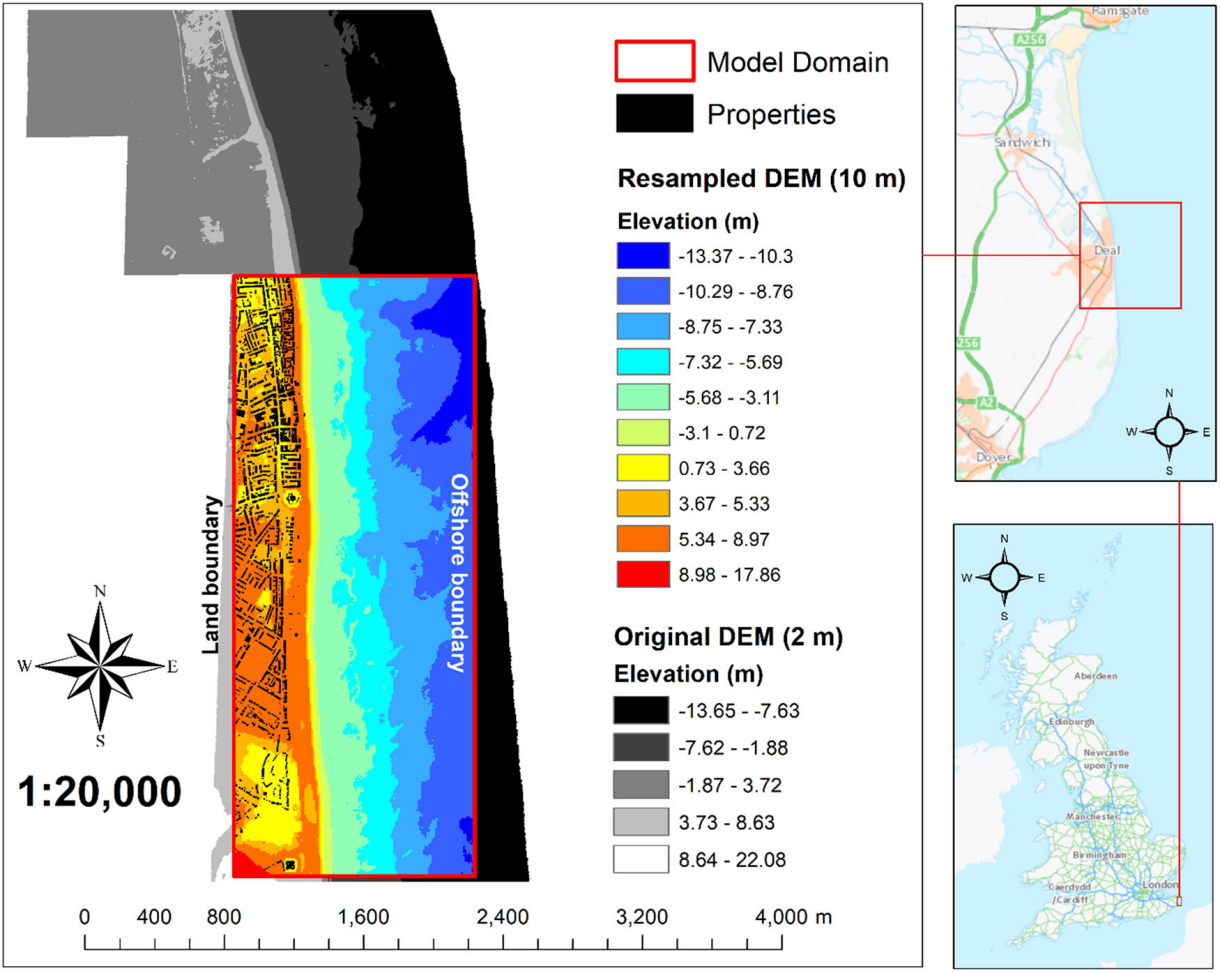
Computational domain and digital elevation models (DEM) used for flood simulations. *Credits*: Ordnance Survey ESRI basemap.

We define a computational domain of 1.4 km (cross‐shore) by 3.5 km (alongshore), interpolated with a 10 m resolution DEM, which was resampled from the United Kingdom 2 m resolution SurfZone 2019 DEM (EA, 2022) using the nearest neighbor approach in ArcGIS10.8.1 (Figure 4). Resampling the SurfZone DEM was necessary to enable computational efficiency. The 10 m resolution used for resampling is the finest, most computationally efficient, spatial resolution that enabled numerical convergence. Importantly, 10 m resolution is fine in relation to the spatial scale of topo‐bathymetric variability at the study site, which exceeds 10 m. We choose the nearest neighbor resampling approach because it is known to preserve high quality values from the original data source (Li & Wong, 2010; Saksena & Merwade, 2015). The DEM used to interpolate the computational domain is vertically referenced to Ordnance Datum Newlyn (ODN) in meters and horizontally referenced to British National Grid, also in meters. The computational domain extends from a land boundary that is ∼6–10 m above ODN to an offshore boundary at a depth of ∼10–13 m below ODN (Figure 4). We use the same computational domain to apply all models.

**FIGURE 5:**
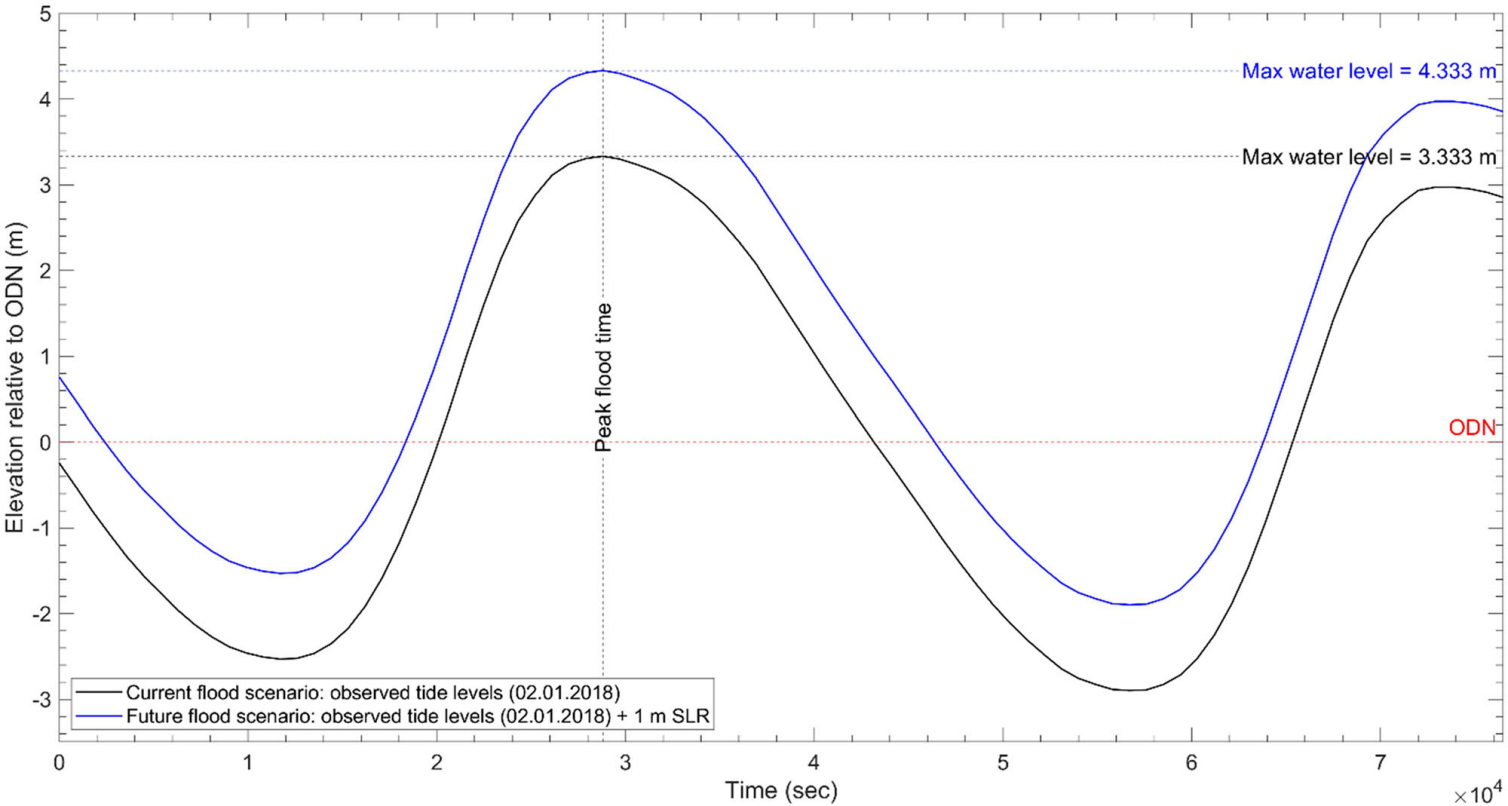
Water levels used to simulate the current and future flood scenario. The current flood scenario is an observed tide surge event from 2018 and the future flood scenario is the same tide surge event superimposed with a 1 m rise in sea‐level (SLR). ODN, Ordnance Datum Newlyn.

We obtain a 21‐h time series dataset of an observed tide surge event that occurred on January 02, 2018 at Dover from the British Oceanographic Data Centre (Figure 5). This dataset is in 15‐min intervals and vertically referenced to ODN. To simulate the current flood scenario, we use this dataset to drive flood propagation in LISFLOOD−FP and the highest water level in this dataset (i.e., 3.3 m above ODN) to apply the BTM. In LISFLOOD−FP, we force the tide data at the offshore boundary in the model domain. We keep the connecting boundaries open to allow flow in and out of the domain. We superimpose a 1 m sea‐level rise onto the 21‐h time series tide data obtained (Figure 5) and use this to simulate the future flood scenario in LISFLOOD−FP. We use the highest water level from this superimposed dataset (i.e., 4.3 m above ODN) to simulate the future flood scenario using BTM.

We run all LISFLOOD−FP simulations over a 21‐h period, typical of tide surge events. Unlike more complex flood models (e.g., TELEMAC−2D), LISFLOOD−FP has only one free parameter—bed friction—based on Manning's n. Generally, the specification of Manning's n is subject to extensive calibration. However, as this is an experimental study designed to understand real estate demand decisions in response to flood predictions, extensive model calibration and validation is not required. To be objective, we ensure that all models: (a) have the same setup and data, and (b) are applied based on established guidelines for flood simulations (Cunge, 2003; Neal et al., 2012; Seenath et al., 2016; Smith et al., 2011). For LISFLOOD−FP simulations, we specify a friction of 0.02—the Manning's n value for open water/sand (Chow, 1959; Garzon et al., 2023; Mattocks & Forbes, 2008; Seenath, 2018)—which broadly characterizes our study location. BTM simulations entailed a rapid calculation procedure in ArcGIS10.8.1 that identified areas in the DEM lower than the highest tide levels in the flood scenarios. Table 1 summarizes all model specifications.

**TABLE 1:** Specifications used to apply the LISFLOOD−FP solvers and the BTM in this paper.

Input	LISFLOOD−ROE	LISFLOOD−ACC	LISFLOOD−FL	BTM
DEM	10 m resampled SurfZone DEM			
bcifile ^a^ spec.	Time‐varying free surface elevation on the east side of the domain between BNG northing coordinates 153,476 and 150,036 m			Not applicable
bdyfile ^b^ spec.	Tide levels in Figure 5			Not applicable
startfile ^c^	10 m resampled SurfZone DEM containing water depth only			Not applicable
sim_time ^d^	76,500 s			Not applicable
saveint ^e^	1000 s			Not applicable
massint ^f^	1000 s			Not applicable
elevoff ^g^	Activated			Not applicable
fpfric ^h^	0.02			Not applicable
initial_tstep ^i^	10 s			Not applicable
Solver	Roe	Acceleration	Flow‐limited	Not applicable
adaptoff ^j^	Not activated	Not activated	Activated	Not applicable
Max. flood level (current scenario)	Not applicable			3.3 m ODN
Max. flood level (future scenario)	Not applicable			4.3 m ODN
Flood calculation (current scenario)	See governing equations in Section 3.1.1			=DEM<3.3mODN
Flood calculation (future scenario)	See governing equations in Section 3.1.1			=DEM<4.3mODN

Abbreviations: DEM, digital elevation model; ODN, Ordnance Datum Newlyn.

^a^ Specification of boundary condition type and coordinates, from which boundary conditions are forced in the model domain.

^b^ Specification of the time‐varying boundary conditions that are forced in the model (in this case, tidal levels).

^c^ Specification of water depth file, providing initial conditions for a simulation.

^d^ Specifies the duration of the simulation in seconds.

^e^ Specifies the interval, in seconds, at which flood results are saved during a simulation. In this case, flood outputs are saved every 1000 seconds in the simulation.

^f^ Specifies the interval, in seconds, at which mass balance data are outputted.

^g^ Suppresses the output of water surface elevation files at each saveint. These files are unnecessarily large and not considered in this paper.

^h^ Specifies the friction value, which takes the form of Manning's n.

^i^ Specifies the initial (warm up) model time step in seconds.

^j^ Suppresses adaptive time stepping algorithm and a fixed time step is used.

#### 3.1.4 Flood maps

We generate two flood maps from each model, one each for the current and future flood scenarios using ArcGIS10.8.1. We use these maps to gauge the uncertainties in flood predictions relative to model complexity and as the basis for our main online survey, which investigates the influence of access to multiple sources of flood model predictions on coastal real estate demand decisions.

We generate the LISFLOOD−ROE, LISFLOOD−ACC, and LISFLOOD−FL flood maps based on their maximum flood depth raster output. To distinguish between flood and non‐flood areas, we apply a depth threshold >0 m using a simple raster calculation equation in ArcGIS10.8.1, following Seenath et al. (2016). Specifically, for each flood scenario, we consider areas with a predicted maximumflooddepth>0m to flood. Using a depth threshold >0 m enables an objective comparison with BTM predictions, as BTM considers all areas lower than the maximum flood water level to be flood vulnerable.

To generate the BTM flood maps, we apply the same raster calculation process outlined above in ArcGIS10.8.1 to identify areas in the resampled DEM (Figure 4) that are lower than the highest flood water level in the current (3.333 m ODN) and future flood (4.333 m ODN) scenarios (Figure 5).

### 3.2 Primary data collection

A novel element of our study involves understanding whether access to multiple sources of flood model predictions can influence coastal real estate demand decisions, measured through: (a) WTP for properties in flood and non‐flood prediction zones, and (b) location preferences. To do this, we adopt a mixed open and closed‐ended reactionary survey, as outlined below.

#### 3.2.1 Survey design

**FIGURE 6:**
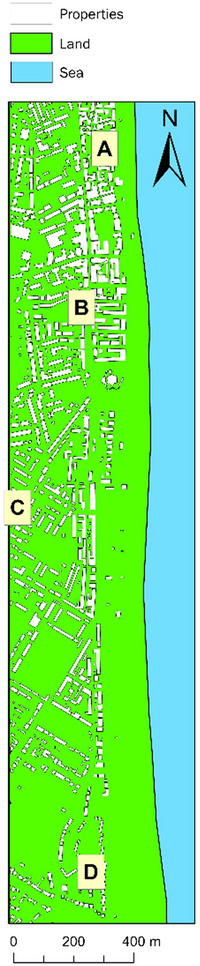
The four locations used for the WTP survey, labeled A–D.

Our survey targets UK residents ≥18 years old and contains 13 questions—one eligibility question and 12 questions based on hypothetical scenarios designed to investigate the unbiased influence of having access to multiple sources of flood model predictions on real estate demand decisions. We first ask respondents to specify the first part of their UK postcode (i.e., eligibility question). We then introduce three scenarios:

1. In *scenario one*, we ask respondents to assume that they are interested in buying or renting a property in a UK coastal town—Deal (Figure 3). Although our scenario focuses on Deal, we do not reveal this location in the survey and instead provide participants with a summary of the key characteristics of the town outlined in Section 2. We do not reveal the town for three reasons: (a) the location is not central to our narrative; (b) our research is experimental and exploratory, designed to gauge the potential influence of having access to multiple sources of flood model predictions on real estate demand decisions; and (c) to avoid panic and distress regarding flood vulnerability, especially for the case study site. In this first scenario, we ask respondents to specify how much they are willing‐to‐pay (WTP) to buy and rent properties in four locations identified as A (commercial seafront area), B (mixed residential and commercial area near the sea), C (residential area away from the sea), and D (secluded seafront residential area) in Figure 6, where WTP = maximum amount of money they are WTP to buy and rent a property. We select these locations based on conflicting flood extent predictions obtained from the models applied (Section 4.1). However, in this baseline (first) scenario, we do not reveal any flood predictions so that we can obtain location preferences and WTP to buy and rent properties in the absence of flood information. We also ask respondents to specify the reason for their choice in order to understand the factors that drive real estate demand decisions in the absence of hazard risk information, such as flood predictions. To facilitate WTP estimations, we reveal that a two‐bedroom house in the town has an average selling price of £275,000 and an average renting price of £975 per month based on the UK Office for National Statistics (ONS, 2023).
2. In *scenario two*, we provide survey participants with flood maps illustrating the current flood scenario predictions from all models applied. Altogether, four flood maps are provided, one each containing the current flood scenario predictions from LISFLOOD−ROE, LISFLOOD−ACC, LISFLOOD−FL, and BTM. We inform all participants that each flood map is based on predictions obtained from different computer models that are commonly used to guide flood management policies in the United Kingdom. We do not provide technical details of the models, nor do we provide information on the flood return period and probability of occurrence. Previous studies show that there is often confusion or failure to understand the technical language that accompany flood maps (Burningham et al., 2008; Henstra et al., 2019). For example, flood experts can easily digest what a “1 in 50 year” flood event means compared to a lay person. Therefore, to avoid confusion related to technical language, we simplify and standardize the presentation of our flood maps to include flood predicted areas in red, non‐flood predicted land areas in green, water bodies in blue, building outlines shaded in white, and a scale bar to indicate distance from the sea. In doing so, we are able to assess the direct influence of access to multiple flood prediction maps on real estate demand decisions. More importantly, our flood mapping and presentation approach enables us to understand whether people can or cannot perceive the uncertainty in flood predictions (evident from conflicting flood maps) in real estate decision‐making, based on their WTP decisions and location preferences. Our approach also allows us to gauge whether real estate decisions are driven by more extreme flood maps (another indication of how people perceive flood risk and its inherent uncertainty). We argue that developing these understandings is the critical *first* step toward improving flood prediction communications before considering the inclusion and presentation of technical information, such as return periods and exceedance probability, in flood communications. In this second scenario, we ask respondents to consider the four flood maps aforementioned and specify how much they will now be WTP to buy and rent properties in the same four locations as before (A, B, C, and D in Figure 6). We also ask them to select their most preferred living location (A, B, C, or D) and to indicate the extent to which they agree that the current flood predictions have influenced their choice of location using a Likert scale (definitely agree, agree, neutral, disagree, and definitely disagree).
3. In *scenario three*, we provide survey participants with flood maps illustrating the future flood scenario predictions from all models applied. Specifically, four flood maps are provided, each containing the future flood scenario predictions from LISFLOOD−ROE, LISFLOOD−ACC, LISFLOOD−FL, and BTM. As before, we inform all participants that each flood map is derived from a different computer model that is commonly used to guide flood management in the United Kingdom. For reasons mentioned earlier, we do not provide any technical information about the models (e.g., functional form) and their predictions (e.g., return periods). In this third scenario, we ask respondents to consider the four future flood maps and specify how much they will now be WTP to buy and rent properties in the same locations as before (A, B, C, and D in Figure 6). Again, we also ask them to select their most preferred living location (A, B, C, or D) and to indicate the extent to which they agree that the future flood predictions have influenced their choice of location using the same Likert scale from scenario two.

As respondents progress through the scenarios, we do not enable them to modify answers to previous scenarios. In this way, we capture the influence of having access to multiple sources of flood model predictions on their WTP from their unbiased perspective. We also do not reveal any information relating to flooding prior to introducing the flood predictions. Instead, to capture unbiased real estate demand decisions primarily based on access to multiple sources of flood predictions and reduce researcher bias, we only inform respondents that our survey aims to understand the factors influencing WTP to buy and rent coastal properties. Our supplementary files include a copy of the survey, which we developed using JISC Online Surveys (https://www.onlinesurveys.ac.uk/).

#### 3.2.2 Pilot testing, dissemination, and data processing

We first pilot the survey to ensure that we can address our research question and then disseminate to United Kingdom residents online. We acquire 572 responses from May 1 to July 31, 2023, including 299 from Prolific (https://www.prolific.com/), 160 from SurveyCircle (https://www.surveycircle.com/en/), 20 from SurveySwap (https://surveyswap.io/), and 93 from other sources, including social media and mailing lists.

We inspect the 572 responses obtained, excluding ineligible and impaired responses. As our survey targets adult residents in the United Kingdom, all respondents that provide an invalid United Kingdom postcode or fail to answer compulsory questions are excluded from our final dataset. Altogether, we obtain 532 usable survey responses to inform our study. Our final survey dataset is included in our Supporting Information.

### 3.3 Statistical and geospatial analyses

#### 3.3.1 Statistical analysis

To answer whether flood predictions affect real estate demand, we examine how participants change their location preferences and WTP decisions for properties in locations A–D in Figure 6 *before* (baseline scenario) and *after* the current and future flood scenario predictions are introduced. Additionally, to investigate whether access to multiple sources of flood model predictions creates more uncertainty in real estate decision‐making, we follow the finance literature and use market volatility to proxy real estate risk. Hence, we compare changes in the standard deviations—a common and simple measure of volatility—in property sale and rental WTP prices in the presence of flood prediction information. We also compute the mean differences in WTP values for buying and renting properties in locations A–D in Figure 6, to determine gains and losses. Using mean differences between WTP values in the baseline and flood scenarios control for participants who, whether because of socioeconomic reasons or personality traits, are inclined to offer discounted or premium WTP values against the average rate of £275,000 in the sale market and £975 in the rental market for a two‐bedroom property in the coastal town considered. It also directly facilitates the estimation of paired sample *t*‐tests to evaluate the statistical significance in changes *before* and *after* flood prediction maps are introduced.

#### 3.3.2 Geospatial analysis

We consider the .max and .maxtm outputs from each LISFLOOD−ROE, LISFLOOD−ACC, and LISFLOOD−FL simulation. The .max outputs indicate the maximum flood depth predicted in each cell of the DEM over the entire simulation. The .maxtm outputs indicate the time of maximum flood depth occurrence in each cell of the DEM over the entire simulation. For each flood scenario, we quantify the spatial differences in flood depth and flood timing predictions from each LISFLOOD−FP solver through raster‐based vertical differencing in QGIS3.16.10. We use the outputs to create two raster‐difference matrices for each flood scenario in ArcGISPro3.1.0, one showing flood depth differences and the other showing flood timing differences. Although these outputs are not central to our core narrative, and although we do not use these matrices in our survey, considering such information alongside our survey data allows us to extrapolate how flood prediction uncertainty may affect the selection of flood evacuation routes, and the potential impacts that this may have on coastal real estate decision‐making.

### 3.4 Robustness study

We recognize that the design of our flood maps, which underpin our primary data collection survey (Section 3.2.1), could potentially skew the decision‐making process of our participants in terms of their property pricing and preferences in response to flood prediction information. For example, our flood maps provide binary options—either an area is predicted to flood (red) or not flood (green)—which may push people toward making extreme, risk averse decisions, by prioritizing risk of flooding over other factors, such as location preferences. Additionally, our decision to omit information on flood probabilities in our binary flood maps may convey a message of “actual” flooding and not flood “risk,” with implications for understanding real estate consumer behavior in the presence of flood “risk” information. Therefore, in addition to our main survey, we run a robustness experiment survey to ascertain whether our results from our hypothetical flood scenarios matches up to reality in terms of how people respond to an actual open‐access flood probability map.

**FIGURE 7:**
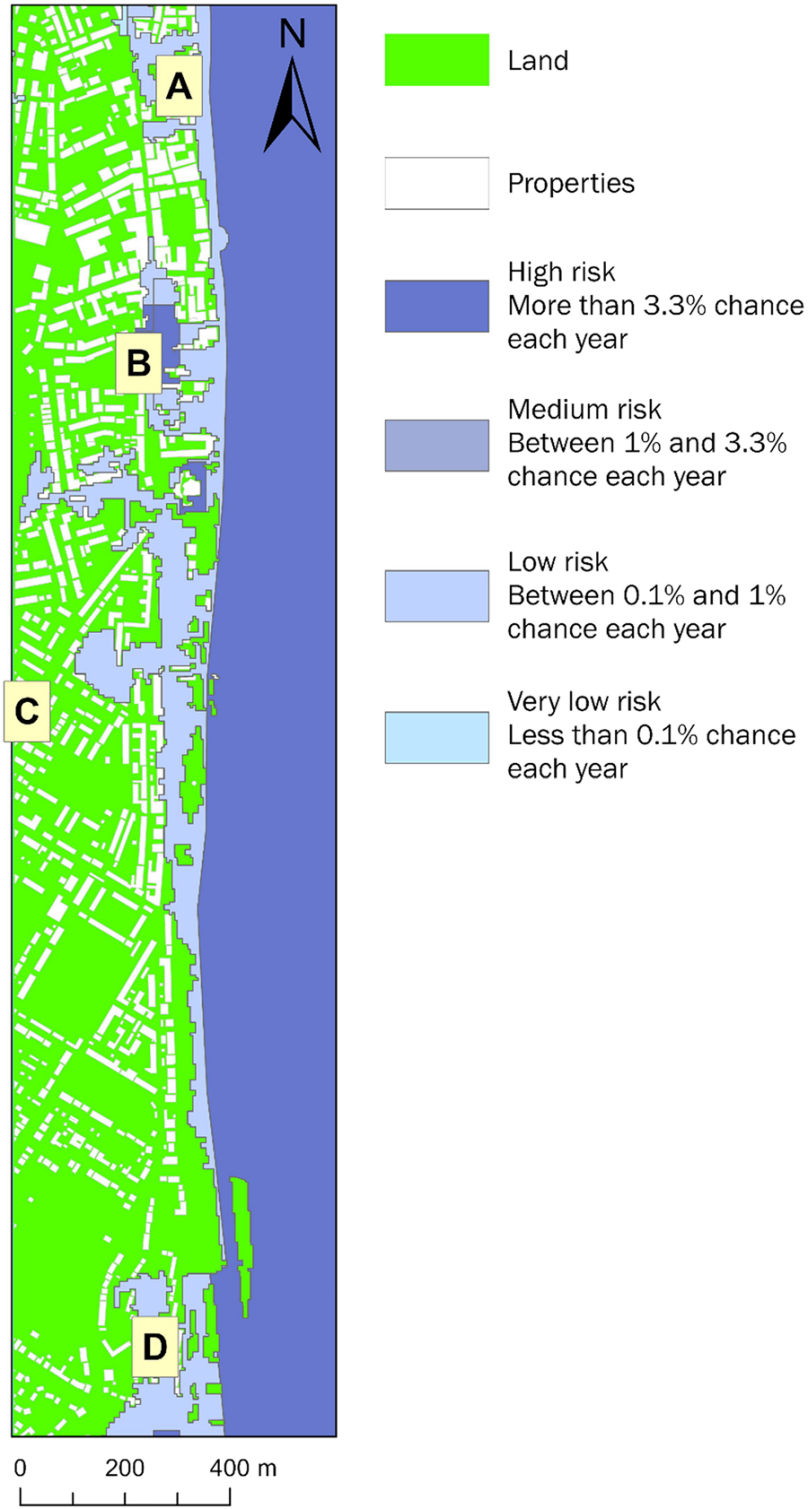
*Risk of Flooding from Rivers and Seas* (RoFRS) long‐term flood risk probability map for the case study site (EA, 2023).

Our robustness experiment survey has the same eligibility questions and flood scenario setup as our main survey (Section 3.2.1). Specifically, in this survey, we first ask respondents to specify the first part of their United Kingdom postcode (eligibility question) and then ask questions around two scenarios—one baseline scenario without flood information and, then, another with flood risk probabilities introduced. The first scenario is exactly the same as the first scenario in our main survey, containing no flood information and asking participants to specify their: (a) WTP to buy and rent properties in locations A, B, C, and D in Figure 6; (b) most preferred living location of the four options presented (A, B, C, or D); and (c) reasons for selecting their most preferred living location (see specific details in Section 3.2.1). In the second scenario, we introduce England's open‐access RoFRS long‐term flood risk predictions for the case study site (Figure 7; EA, 2023) and ask participants to consider this information and update their: (a) WTP to buy and rent properties in the same four locations as before (A, B, C, and D in Figure 6) and (b) most preferred living location (A, B, C, or D). We also ask them to indicate the extent to which they agree that the long‐term flood risk information has influenced their choice of location using a Likert scale identical to the main survey design. To be consistent with our main survey, we do not enable participants to modify answers as they progress through the two scenarios so that we capture the influence of having access to the long‐term flood risk probability information on their WTP from their unbiased perspective. We also do not reveal any information relating to flooding prior to introducing the open‐access long‐term flood risk probability predictions from the RoFRS model. Instead, to capture unbiased real estate demand decisions primarily based on access to such predictions and reduce researcher bias, we only inform respondents that our robustness survey aims to understand the factors influencing WTP to buy and rent coastal properties. Our supplementary files also include a copy of the robustness survey experiment.

We disseminate our robustness survey to UK residents online, using Prolific, ensuring that Prolific participants that previously completed our main survey are excluded from the robustness experiment survey. Doing so ensures that all participants had no pre‐conceived notions about the survey to reduce any participant bias associated with prior knowledge of the main survey. We obtain 202 responses to our robustness survey and, following data inspection for impaired or invalid responses, we end up with 199 usable responses. We include the robustness survey data in our supplementary materials and apply the same methods outlined in Section 3.3.1 to analyze this data.

Our robustness survey experiment allows us to validate our findings on whether: (a) people can perceive the uncertainty in flood risk information when making real estate demand decisions; and (b) access to flood prediction information presents a risk to the real estate market. Our robustness study also enables us to examine whether alternative modes of flood prediction communication (binary flood maps versus flood risk probability maps) affect residential real estate decision‐making.

## 4 RESULTS AND ANALYSIS

### 4.1 Flood outputs

**FIGURE 8:**
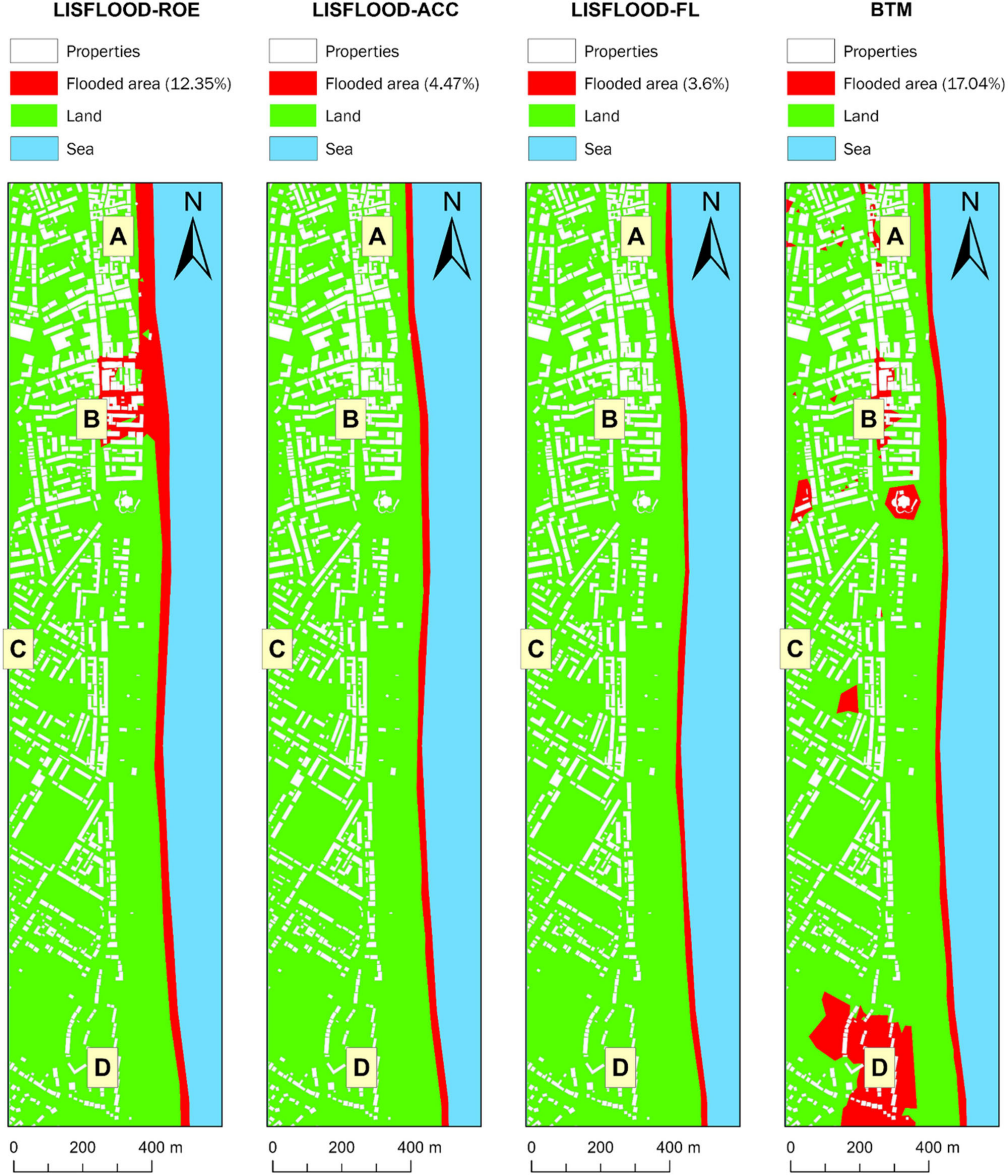
LISFLOOD−FP and the BTM predictions of flood extent under the current flood scenario.

**FIGURE 9:**
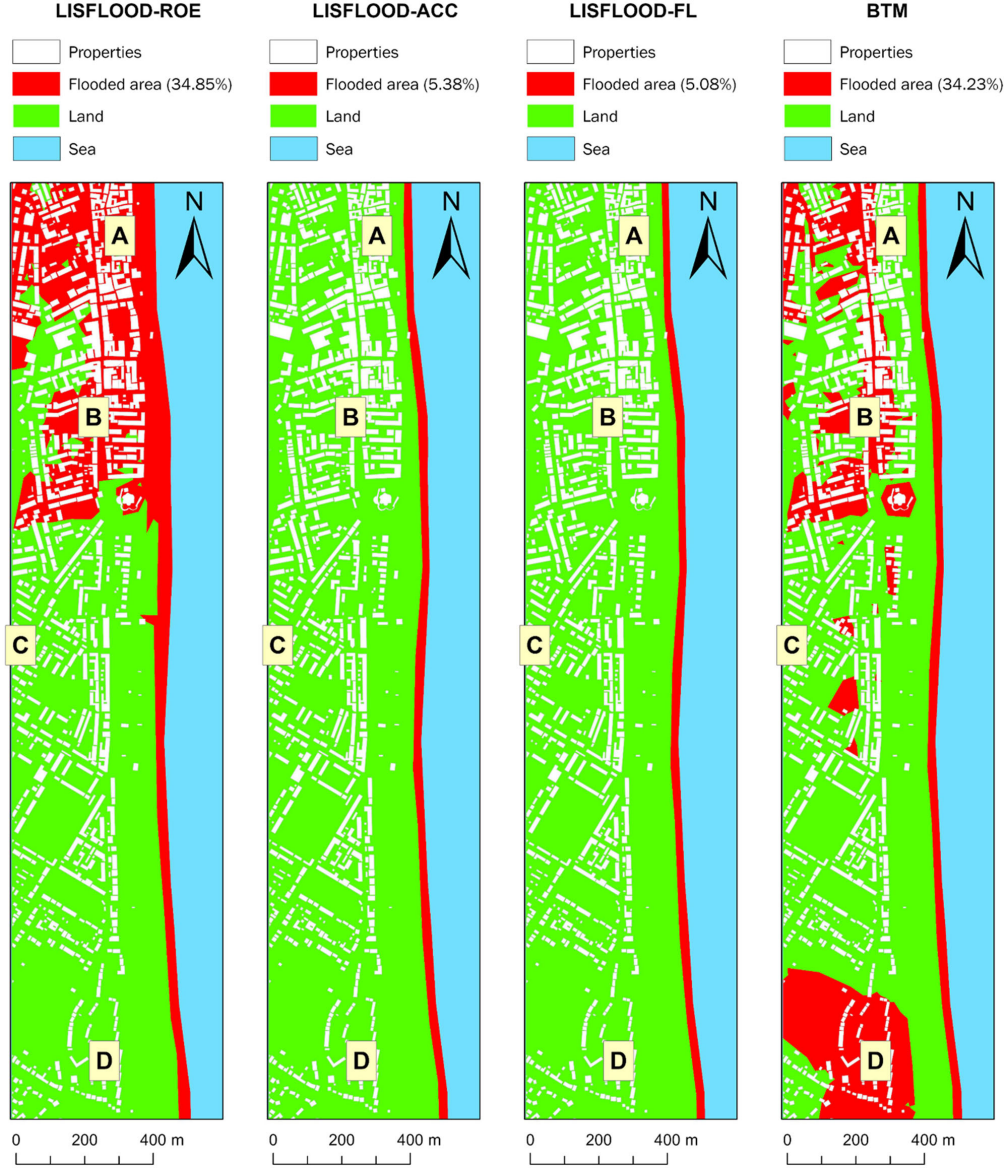
LISFLOOD−FP and the BTM predictions of flood extent under the future flood scenario.

Figures 8 and 9 illustrate the flood predictions from all models for the current and future flood scenarios, respectively. Table 2 summarizes the differences in flood predictions shown in Figures 8 and 9 in relation to the four locations in Figure 6, in order to gauge flood prediction uncertainty relative to model structure.

**TABLE 2:** Flood predictions for locations A—D in Figure 6.

Location	Flood predictions
**A**	⇒ Not predicted to flood under the current and future flood scenarios from LISFLOOD−ACC and LISFLOOD−FL. ⇒ Only predicted to flood under the future flood scenario from LISFLOOD−ROE. However, under the current flood scenario, LISFLOOD−ROE predicts flood propagation up to location A, implicitly indicating that this location will flood with further rises in flood water level. ⇒ Under the future flood scenario, BTM predicts that areas immediately west of location A will flood. A caveat here is that these flood areas predicted from BTM are not hydraulically connected to those at the coast.
**B**	⇒ Not predicted to flood under the current and future flood scenarios from LISFLOOD−ACC and LISFLOOD−FL. ⇒ Predicted to flood under the current and future flood scenarios from LISFLOOD−ROE and BTM. However, yet again, we see that flood predicted areas from BTM are not hydraulically connected.
**C**	⇒ Not predicted to flood under the current and future flood scenarios from LISFLOOD−ACC and LISFLOOD−FL. ⇒ Not predicted to flood under the current and future flood scenarios from all models.
**D**	⇒ Not predicted to flood under the current and future flood scenarios from LISFLOOD−ACC and LISFLOOD−FL. ⇒ Predicted to flood under the current and future flood scenarios from BTM. However, we find that areas predicted to flood inland and at the coast from BTM are not hydraulically connected, in line with findings in related literature (Leijnse et al., 2021; Seenath et al., 2016; Williams & Lück‐Vogel, 2020).

Collectively from the information in Figures 8 and 9 and Table 2, we observe that all models agree that location C is not vulnerable to flooding under the current and future flood scenarios, consistent with England's RoFRS long‐term flood risk predictions, which also show that location C is not at risk of flooding (Figure 7). However, this agreement is not indicative of convergence between model structures but instead is due to the elevation of location C (i.e., ∼6 m above ODN) exceeding the maximum flood water level in each scenario (Figure 5). The agreement between models on location C's flood vulnerability is, therefore, not an indication of model consistency or reduced uncertainty. Conversely, there is disagreement between (LISFLOOD−FP versus BTM) and within (LISFLOOD−FP solvers) models with respect to whether locations A, B, and D are vulnerable to flooding under both flood scenarios. We also see that LISFLOOD−ROE and BTM predict a notably larger inland extent of flooding than LISFLOOD−ACC and LISFLOOD−FL. The key difference is that flood predictions from LISFLOOD−ROE are hydraulically connected as opposed to those from BTM. LISFLOOD−ACC and LISFLOOD−FL predictions are consistent in each flood scenario despite LISFLOOD−ACC predicting a slightly larger inundated area. The spatial extent of flooding predicted from LISFLOOD−ACC and LISFLOOD−FL in each flood scenario is confined to the beach and does not include inland areas. This corresponds well with the beach profile at the site (Figure 3B), which shows a steep upper beach that will likely act as a natural flood defense against inland flooding. These differences in flood predictions are all indicative of flood prediction uncertainty linked to model structure, which may influence WTP values for buying and renting properties in the four locations considered. WTP decisions in this regard will likely depend on the ability of people to recognize the uncertainty in these predictions, their level of risk aversion, and their flood experiences and awareness.

In the absence of model validation, we use deductive reasoning to make inferences on which of the four models are most accurate based on: (a) physical site characteristics (Figure 4) and (b) the maximum flood water levels simulated in each scenario (Figure 5). In the case of BTM, the inland predictions of flooding are erroneous and do not conform to flood routing physics as the inland flooded areas are not connected to the flooded areas at the coast (Figures 8 and 9). This is indicative of BTM's inability to account for hydraulic connectivity and flood routing physics, which often leads to flood extent overestimation (Leijnse et al., 2021; Seenath et al., 2016; Williams & Lück‐Vogel, 2020). The inland flood predictions from LISFLOOD−ROE are also erroneous as the beach berm elevation alongshore falls within the range of 6–8 m above ODN (Figure 4), which is higher than the maximum water level simulated under the current (3.33 m above ODN) and future (4.33 m above ODN) flood scenarios (Figure 5). This means that beach berm overtopping and associated inland flooding predicted by LISFLOOD−ROE are not physically realistic. Interestingly, we see that A and D have a low long‐term flood risk based on the RoFRS predictions that are open‐access in England, whereas B is in a zone with both low and medium flood risk (Figure 7). However, a distinguishing feature of the RoFRS model relative to our models is that the RoFRS considers risk of flooding from both rivers and sea, whereas our models consider flooding from the sea only. This raises an important issue—some models account for one or more types of flooding, which contributes to the list of conflicting flood information sources available to real estate consumers. Nonetheless, we see some areas of convergence between our predictions from LISFLOOD−ROE and BTM and the flood risk probability estimates from the RoFRS model (Figure 7). LISFLOOD−ACC and LISFLOOD−FL predictions are theoretically realistic as their flood predictions are confined to the coast, in areas that are: (a) below the level of the beach berm and (b) lower in elevation than the maximum water level simulated (Figures 4, 5 and 8, 9). However, LISFLOOD−ACC and LISFLOOD−FL make several simplifying assumptions (Section 3.1.1), which do not fully capture the complexity of flood physics. Therefore, although their outputs are theoretically realistic, we need to be cautious that we are not obtaining the “right” outputs for the wrong reasons, where *right* is theoretically realistic predictions and *wrong* is a physically unrealistic model structure representing flood dynamics (i.e., equifinality).

### 4.2 Survey and robustness study results

Table 3 provides unambiguous support that flood predictions (both binary predictions and risk probability estimates) affect property location preferences. The relatively more popular property location choices selected by respondents prior to the introduction of flood predictions in our main survey are locations A (36%) and D (30%), whereas B (18%) and C (16%) are less popular. However, C becomes the most popular choice for buying and renting properties when the current (60%) and future (75%) flood predictions are introduced. These results are consistent with those from our robustness study, which show that: (a) locations A (28%) and D (41%) are most popular relative to B (17%) and C (14%) before the RoFRS long‐term flood risk probabilities are introduced, and (b) location C (58%) becomes the most preferred location after flood probabilities are introduced (Table 3).

**TABLE 3:** Location preference under alternative hypothetical scenarios.

			Flood scenarios				Robustness study			
	Baseline		Current		Future		Baseline		RoFRS predictions	
Location	*Freq*.	*Percent*	*Freq*.	*Percent*	*Freq*.	*Percent*	*Freq*.	*Percent*	*Freq*.	*Percent*
**A**	189	36	129	24	57	11	55	28	36	18
**B**	96	18	31	6	17	3	34	17	5	3
**C**	86	16	317	60	397	75	28	14	116	58
**D**	161	30	55	10	61	11	82	41	42	21
	532	100	532	100	532	100	199	100	199	100

*Note*: Freq. is the frequency and it is counted in number of respondents. RoFRS predictions is the England's long‐term flood risk predictions from rivers and sea (EA, 2023).

From our main survey, we find that close proximity to the sea is the most cited reason for the selection of locations A and B as preferred living locations in the absence of flood information, whereas it is safety against hazard risk and seclusion for C and D, respectively (Table 4). Access to amenities (convenience) and seafront views (location esthetics) are other popular reasons for location preferences (Table 4). Safety, quite interestingly, only appears to become a noteworthy driver of real estate decisions for properties in the inland locations—B and C (Table 4), potentially signaling a risk averse group of participants in our study. These findings are also consistent with those from our robustness study (Table 4). The overall shift to C as the preferred living location after flood information is introduced strongly suggests that safety becomes the deciding factor, taking precedence over personal prior preferences, of real estate decisions when such information is made available.

**TABLE 4:** Factors influencing location preferences preflood information.

		Specified reasons for preferred location choice (%)—main survey							
Location	Freq.	Views	Convenience	Close proximity to sea	Seclusion	Safety	Cost	Neighborhood	Others
**A**	189	20.1	19.0	**70.4**	1.6	1.1	1.1	1.6	1.1
**B**	96	3.1	32.3	**46.9**	1.0	17.7	2.1	0.0	2.1
**C**	86	1.2	5.8	2.3	4.7	**65.1**	2.3	1.2	2.3
**D**	161	10.6	2.5	13.7	**61.5**	6.8	1.2	1.9	3.1

		Specified reasons for preferred location choice (%)—robustness study							
Location	Freq.	Views	Convenience	Close proximity to sea	Seclusion	Safety	Cost	Neighborhood	Other
**A**	55	10.9	18.2	**78.2**	–	–	–	21.8	–
**B**	34	11.8	26.5	**64.7**	–	29.4	14.7	32.4	2.9
**C**	28	–	10.7	–	**–**	**57.1**	7.1	35.7	10.7
**D**	82	12.2	2.4	41.5	**78**	8.5	1.2	3.7	7.3

*Note*: Convenience is the access to amenities. Safety is the “safety against flood risk.” Freq. is the total number of survey participants selecting A, B, C, or D as their preferred living location. Each row and column of percentages do not add up to 100% as individual participants often quoted more than one reason for their selection of a preferred living location. The percentage values listed for a specific reason (“Views,” “Convenience,” etc.) under a specific location (A, B, C, or D) is the total number of times that reason has been quoted for the selection of that location/total number of all quoted reasons for the selection of that location × 100.

Additionally, findings from our main study reveal that the highest mean WTP buying and renting values in the *baseline* scenario (non‐flood scenario) are associated with location A, the only location that has an average WTP price above the ONS average property sale and rental values of £275,000 and £975, respectively (Table 5). In the baseline scenario, locations B and D have similar average WTP values of ∼ £10,000 (buying) and £30 (rental) below the ONS average values. C, on the other hand, has the lowest average WTP value of all locations at < £20,000 (buying) and £85 (rental) below the ONS average values. Yet, C switches from having the lowest to the highest WTP buying and rental values under both flood scenarios, whereas all other locations record considerable drops in WTP buying and rental values when flood information becomes available. Moreover, the elevated standard deviation values across all WTP buying and renting prices after flood prediction scenarios are provided, when compared to the baseline scenario, indicate that flood prediction information increases the uncertainty in real estate demand decisions, presenting heightened risks for the real estate sector. These findings match those from our robustness study, which shows: (a) location A attracts WTP buying and renting values above the corresponding ONS estimated values before flood risk probabilities are introduced; (b) B and D attract WTP buying and renting values slightly below the corresponding ONS estimated values; and (c) C attracts the lowest WTP buying and rental values. After flood risk probabilities are introduced, locations A, B, and D record considerable drops in WTP buying and rental values, whereas C attracts the highest WTP buying and renting values (Table 5). This is a particularly interesting finding as two different approaches for communicating flood risk—binary flood maps (non‐flood and flood—main survey) and flood risk probabilities (likelihood of flood risk—robustness study)—resulted in replicated real estate market behavior. This finding implies that people perceive flood communications through a binary lens—whether or not a property would flood—without considering the uncertainties (evident from conflicting flood prediction sources in the main survey) and probabilities in flood risk communications.

**TABLE 5:** Willingness to buy and rent coastal properties under alternative scenarios.

					Flood scenarios							
	Baseline				Current				Future			
	For sale (£)		To rent (£)		For sale (£)		To rent (£)		For sale (£)		To rent (£)	
Locations	Mean	SD	Mean	SD	Mean	SD	Mean	SD	Mean	SD	Mean	SD
**A**	275,415	73,806	1003	236	243,842	79,141	901	263	209,793	87,709	812	293
**B**	265,709	51,548	946	172	217,784	75,830	816	243	200,470	82,213	784	266
**C**	253,672	40,085	890	154	261,297	48,974	934	433	264,111	53,988	934	191
**D**	264,096	62,347	945	203	217,560	81,205	806	266	215.960	82,526	815	272

	Robustness study							
	Baseline				RoFRS predictions			
	For sale (£)		To rent (£)		For sale (£)		To rent (£)	
Location	Mean	SD	Mean	SD	Mean	SD	Mean	SD
**A**	286,886	65,955	1013	231	247,789	71,927	890	256
**B**	269,098	46,728	936	165	200,317	80,070	751	275
**C**	253,040	42,640	879	154	264,515	51,163	924	165
**D**	273,005	54,608	955	186	246,033	63,247	874	225

*Note*: SD is the standard deviation. RoFRS predictions is the England's long‐term flood risk predictions from rivers and sea (EA, 2023).

Furthermore, Table 6 records the computation of the mean differences between the baseline and flood prediction scenarios from the main survey data. Real estate losses in locations A, B, and D, in both the sale and rental markets, are recorded, whereas gains are observed for properties in C only. We find the same trends in our robustness study (Table 6). Interestingly, the losses incurred through WTP buying and renting values for properties in locations A, B, and D, as well as the gains accrued to properties in location C between the baseline and current flood scenarios, become more pronounced when future flood predictions are introduced in the main survey. For example, in the case of location A, the losses more than double from £31,573 to £65,622. As two out of the four models (LISFLOOD−ROE and BTM) predict a clear increase in the area flooded from the current to future flood scenarios (Figures 8 and 9), there is a notable decline in WTP to buy and rent properties in locations projected to flood (A, B, and D) and an increase in WTP to buy and rent properties in locations outside the flood zone (C). This finding indicates that people are likely to update their decisions in the presence of flood prediction information, consistent with the findings from our robustness study (Tables 3, 5 and 6). For instance, Table 7 conveys that over 80% of participants in the main survey agree that flood predictions—current and future—influenced their location preferences, consistent with the findings from our robustness study, which show that 80% of participants agree that the RoFRS flood risk probabilities influenced their location preferences. Only a minority (< 20%) remain neutral or unconvinced by the flood prediction maps, again consistent with the findings from our robustness study, which also show that 20% remain neutral or disagree with the statement that the RoFRS flood risk probabilities influenced their real estate decisions. Paired sample *t*‐test for comparing the mean differences in WTP to buy and rent between the baseline and flood scenarios in both the main survey and robustness study are highly statistically significant (*p *< 0.01) for all locations (A–D) (Table 6).

**TABLE 6:** Paired sample *t*‐test for mean differences in WTP between the baseline and coastal flood scenarios.

	Paired sample *t‐*test between baseline and coastal flood predictions											
	Current flood scenario				Future flood scenario				Robustness study—RoFRS predictions			
	For sale		To rent		For sale		To rent		For sale		To rent	
Locations	Diff. (£)	*t*‐stat.	Diff. (£)	*t*‐stat.	Diff. (£)	*t*‐stat.	Diff. (£)	*t*‐stat.	Diff. (£)	*t*‐stat.	Diff. (£)	*t*‐stat.
**A**	31,573	11.376^a^	101	11.659^a^	65,622	18.817^a^	190	16.945^a^	39,097	8.870^a^	123	7.847^a^
**B**	47,924	16.534^a^	130	14.407^a^	65,239	19.578^a^	162	15.548^a^	68,781	12.999^a^	184	11.160^a^
**C**	−7624	−4.104^a^	−43	−2.462^a^	−10,438	−5.030^a^	−44	−30.875^a^	−11,475	−3.173^a^	−45	−4.311^a^
**D**	46,536	14.854^a^	139	13.674^a^	48,136	14.967^a^	130	12.844^a^	26,972	6.138^a^	81	5.568^a^

*Note*: Diff. is the difference, and *t*‐stat is *t*‐statistic. The null hypothesis of the paired samples *t*‐test is that there is no difference between the willingness‐to‐pay for a coastal property in the baseline scenario and a given hypothetical coastal flood prediction scenario.

^a^denotes statistical significance of the *t‐*test statistic value at the 1% level, evaluated against Student's *t*‐distribution. Positive (negative) *t‐*test values imply a right (left) tailed hypothesis test is used. RoFRS predictions is the England's long‐term flood risk predictions from rivers and sea (EA, 2023). In the main and robustness surveys, WTP data in the baseline scenarios refer to sale and rental values recorded under their respective non‐flood scenarios (before flood risk information is introduced).

**TABLE 7:** Agreeability that flood predictions influenced location preference.

	Flood scenarios				Robustness study	
	Current		Future		RoFRS predictions	
Responses	Freq.	Percent	Freq.	Percent	Freq.	Percent
Definitely agree	308	58	291	55	105	53
Mostly agree	143	27	142	27	54	27
Neither agree nor disagree	30	6	36	7	12	6
Mostly disagree	34	6	45	8	26	13
Definitely disagree	17	3	18	3	2	1

*Note*: Freq. is the frequency and it is counted in number of respondents. RoFRS predictions is the England's long‐term flood risk predictions from rivers and sea (EA, 2023).

A particularly interesting observation from the main survey is that WTP values for buying and renting properties, in all four locations, are dependent on flood predictions from LISFLOOD−ROE and BTM only. These models show more volatility in flood vulnerability between the current and future flood scenarios, and also predict considerably larger flood extents than LISFLOOD−ACC and LISFLOOD−FL. LISFLOOD−ACC and LISFLOOD−FL predictions are consistent in each flood scenario, indicating that all four locations are safe (Figures 8 and 9). This implies that WTP decisions are especially sensitive to more extreme flood predictions.

## 5 DISCUSSION

Our study shows that there are likely to be considerable real estate risks associated with access to multiple sources of flood prediction information, evident from our survey respondents’ willing‐to‐pay more to live in locations that are not in flood predicted zones (despite the erroneousness and uncertainty in these predictions—Figures 8 and 9) instead of locations that align with their personal preferences (e.g., closeness to sea and convenience) (Tables 3, 5–7). Our robustness study validates these findings, showing that people are willing‐to‐pay more to live in locations that are not associated with any levels of flood risk probabilities (Tables 3, 5–7). These findings indicate that access to flood prediction and probability information can steer real estate demand decisions toward risk aversion, consistent with the findings of Shr and Zipp (2019). However, risk aversion in the context of our study could be attributed to several factors, including flood experiences and awareness as well as coastal residency. Flood experiences and awareness have often been linked to falling real estate demand (property devaluation) in the immediate period following a flood event (Atreya & Ferreira, 2015; Beltrán et al., 2019; Morgan, 2020). However, the empirical real estate literature shows that real estate demand reverts to preflood event levels as time passes, and memories and awareness of such events eventually fade, with personal preferences (e.g., location aesthetics) gradually returning to the forefront of real estate decisions (Atreya & Ferreira, 2015; Beltrán et al., 2019; Bin & Landry, 2013; Bin & Polasky, 2004; Morgan, 2020; Pommeranz & Steininger, 2020). Unlike flood experiences and awareness, which may fade with time, flood risk prediction maps—both binary and probability—are now becoming a permanent feature of online flood risk communications (Figure 2). These predictions are, therefore, likely to have a more enduring impact on real estate demand decisions and overshadow personal preferences (e.g., location esthetics).

Furthermore, although flood memories and awareness are likely to fade with time for those who have experienced a single or a few flood events, the same may not be true for current and past coastal residents whose lived realities involve first‐hand experiences of dealing with coastal hazards that become engrained in memory and have inculcated a culture of risk averse decisions. Such residents are more likely to plan on selling their homes (Bakkensen & Barrage, 2022; Laino & Iglesias, 2023), a decision that can become further forced by having access to multiple flood prediction and risk probability maps. As understanding the data generating process behind our WTP survey experiments is beyond the scope of this study (which only attempts to gauge whether there are potential real estate risks with having access to multiple sources of flood predictions), an important avenue for future research is to investigate the drivers of real estate demand decisions in response to access to flood prediction information. The findings of such research will be pivotal to guide how we frame flood risk communications, especially to lay persons, to reduce real estate risks associated with flood predictions.

**FIGURE 10:**
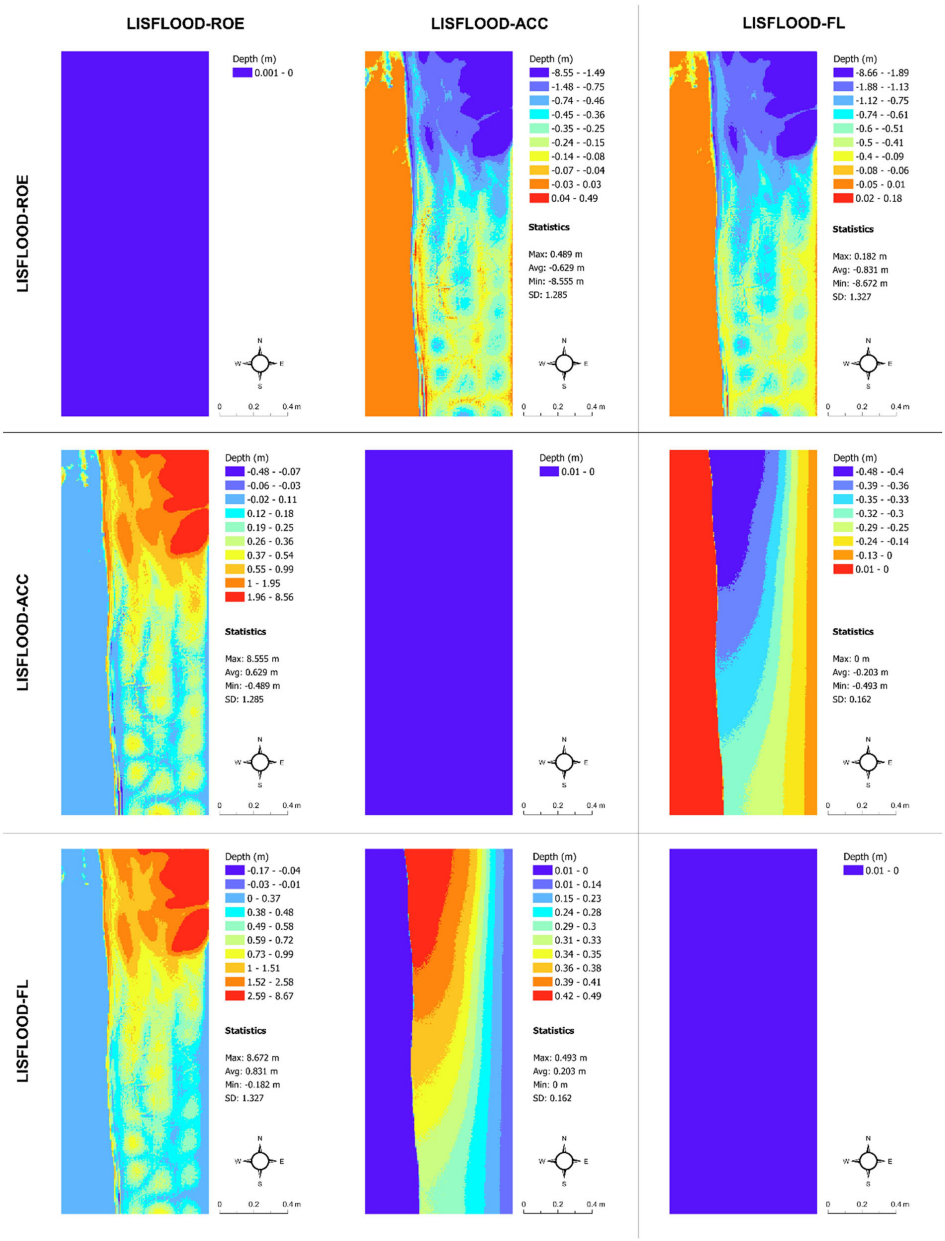
Matrix map of maximum flood depth predictions under the current flood scenario from LISFLOOD−ROE, LISFLOOD−ACC, and LISFLOOD−FL. SD, standard deviation.

**FIGURE 11:**
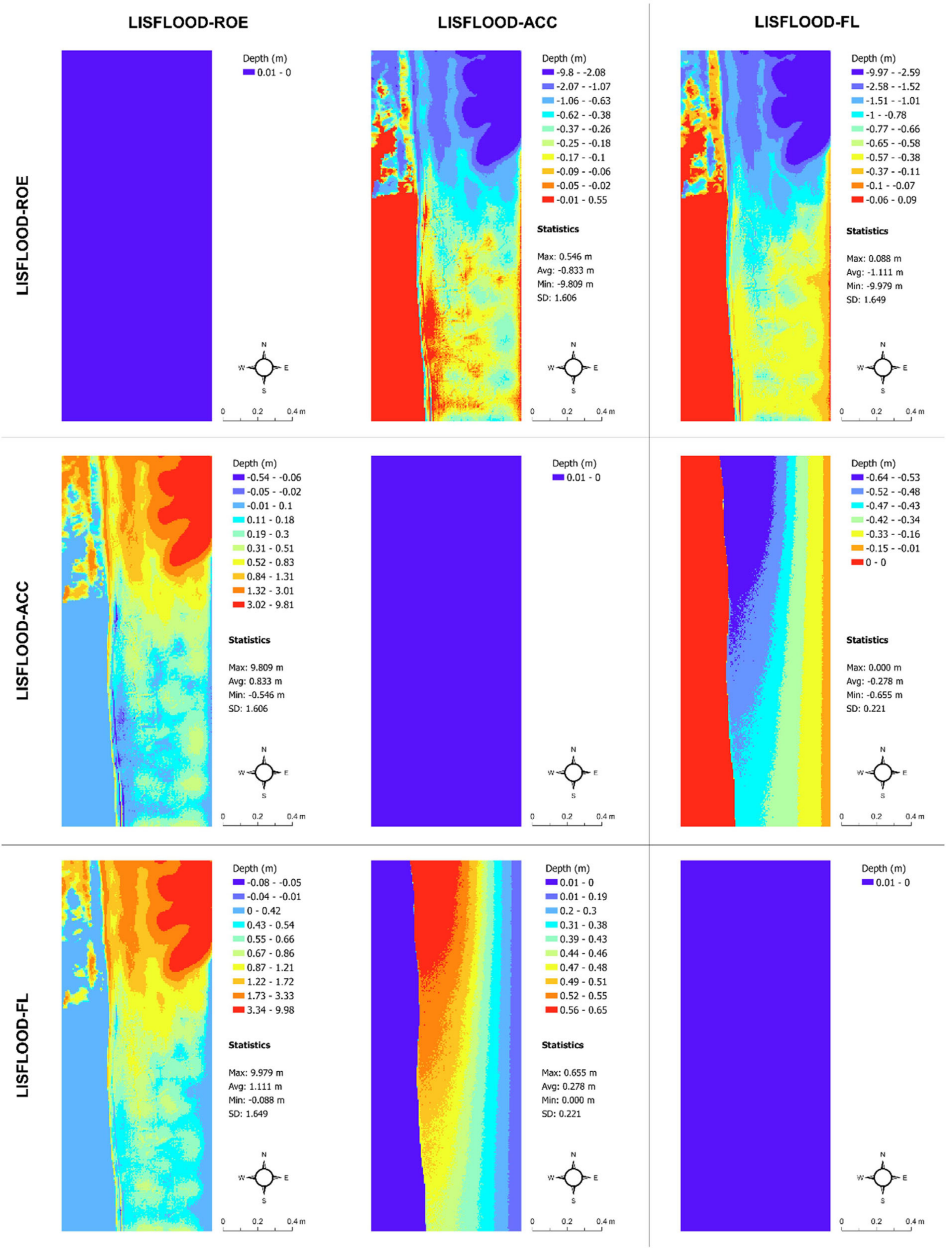
Matrix map of maximum flood depth predictions under the future flood scenario from LISFLOOD−ROE, LISFLOOD−ACC, and LISFLOOD−FL. SD, standard deviation.

**FIGURE 12:**
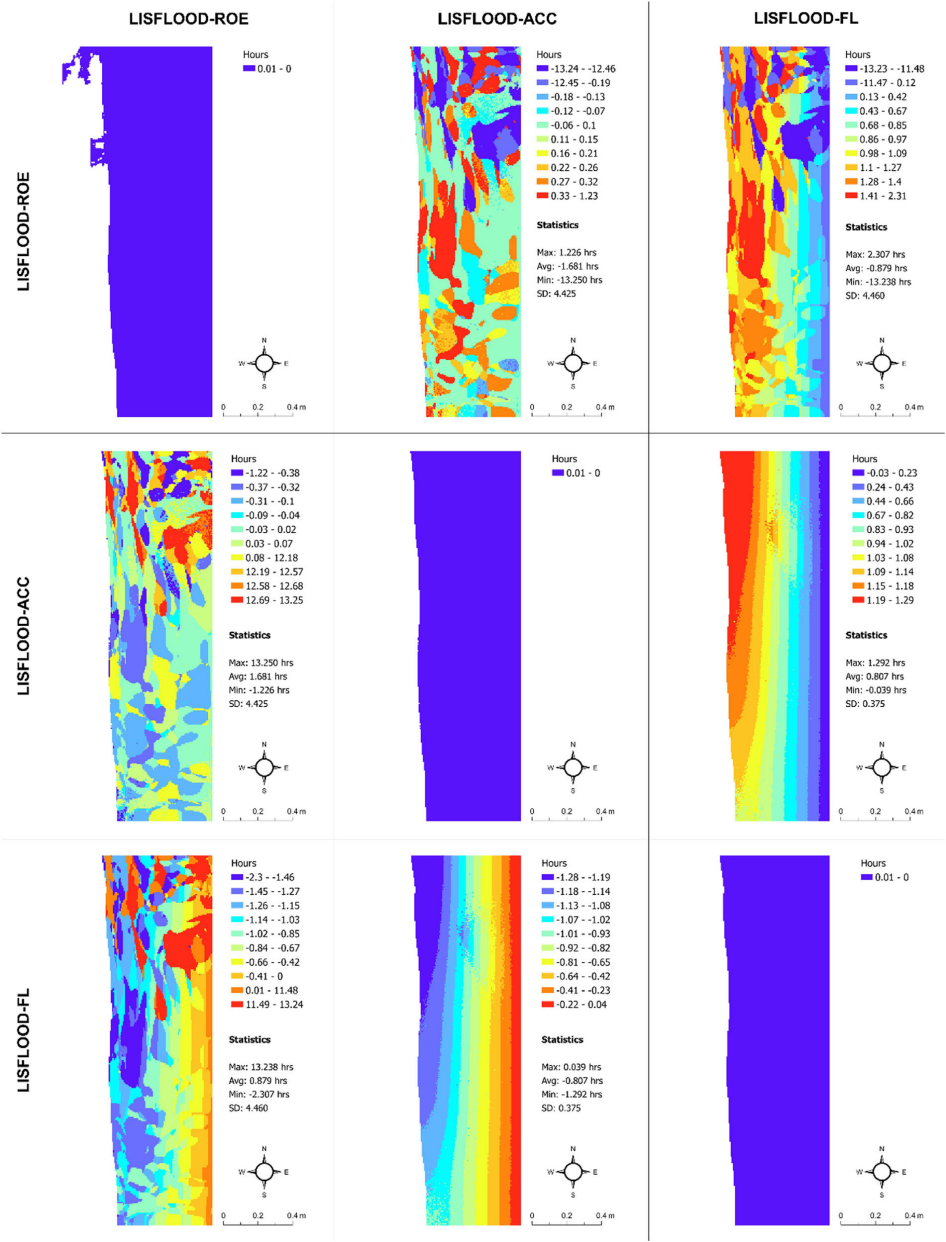
Matrix map of maximum flood timing predictions under the current flood scenario from LISFLOOD−ROE, LISFLOOD−ACC, and LISFLOOD−FL. SD, standard deviation.

**FIGURE 13:**
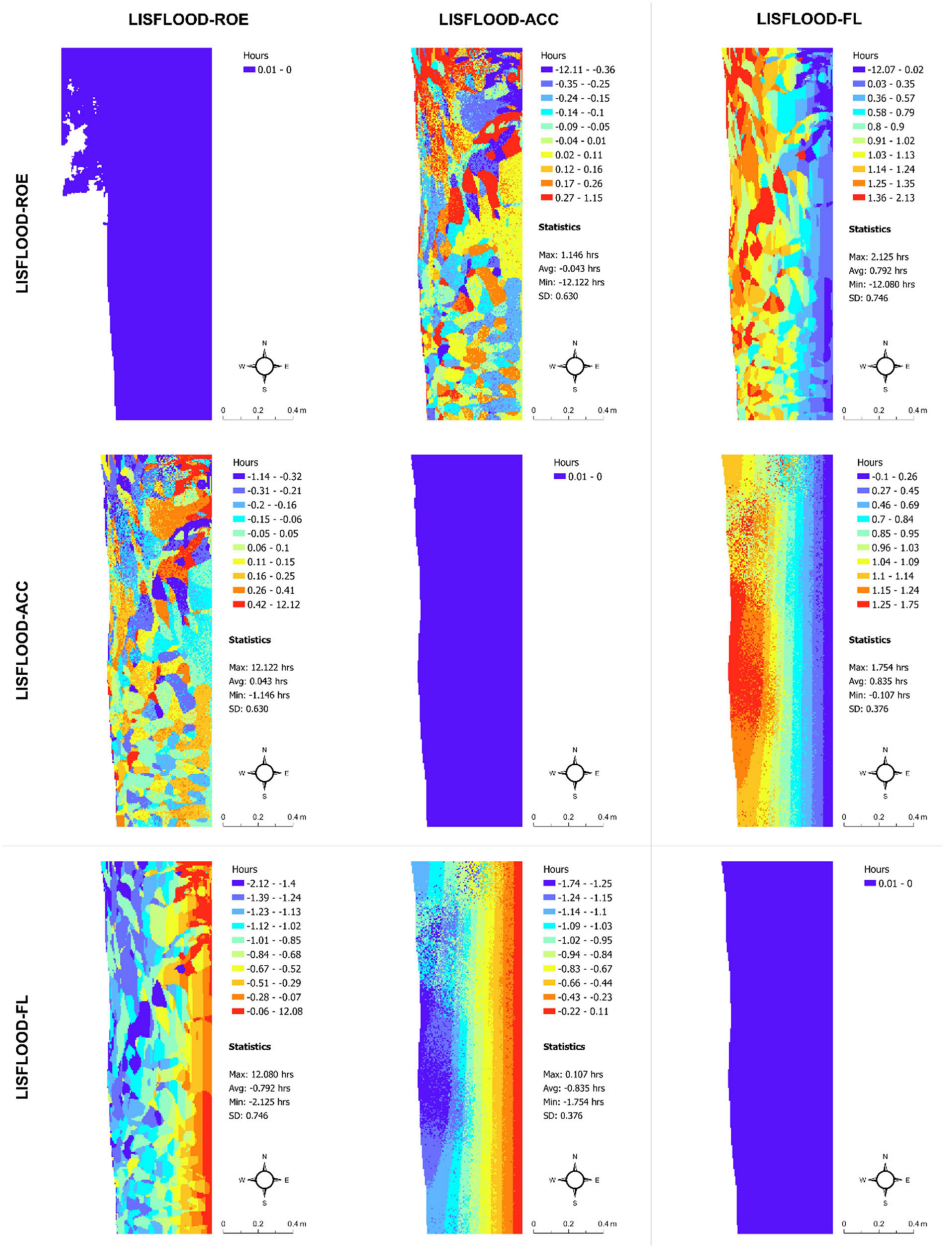
Matrix map of maximum flood timing predictions under the future flood scenario from LISFLOOD−ROE, LISFLOOD−ACC, and LISFLOOD−FL. SD, standard deviation.

Although our study focuses explicitly on flood extent prediction uncertainty (main survey and associated flood models) and flood probabilities (robustness study) and their connection to real estate decision‐making, we also see clear differences in flood depth and timing predictions from the LISFLOOD−FP solvers under the current and future flood scenarios, which underpin our main survey (Figures 10, 11, 12, 13). This is another good example of uncertainty within flood models. Specifically, we see in Figures 10 and 11 that differences in flood depth predictions from LISFLOOD−ACC and LISFLOOD−FL are small compared to the differences in flood timing predictions between these solvers and LISFLOOD−ROE under the current and future flood scenarios. The same is true for the flood timing differences in Figures 12 and 13. Although we do not consider these data in this paper, uncertainties in flood timing and depth predictions can also adversely affect real estate demand decisions as such information often informs flood evacuation routes (Seenath et al., 2016), and properties adjacent to these routes tend to suffer from lower values as people are not keen to live near potential flood risk areas (Hallstrom & Smith, 2005).

Within the context above, we argue that the risk of falling real estate demand in an era of accessible flood prediction and probability information is one that needs to be addressed, as there are considerable risks attached to property devaluation. For instance, the real estate market is an important indicator of macroeconomic performance. This market involves many stakeholders such as estate agents, banks, insurance companies, policymakers, and local communities, and property price fluctuations can impact the expected lifetime wealth of homeowners and the collateral values of homes (Rajapaksa et al., 2016). Therefore, properties perceived to have an elevated exposure to the risk of natural hazards not only affect homeowners but can have knock‐on effects for financial institutions and government policy. How individuals perceive risk, as well as their inability to distinguish between assessed and perceived risk, remain revolving issues in hazard and flood management (Atreya & Ferreira, 2015). Similar to Atreya and Czajkowski (2016), we find that seafront locations, such as location A, attract price premiums (Table 3), primarily for reasons relating to close proximity to the sea (location aesthetics) (Table 4). This perception changes when flood prediction information becomes available, as respondents show stronger preferences for properties in location C—perceived as flood safe—in terms of preferred location choice and WTP more to buy and rent here (Tables 3, 4, 5, 6). Moreover, our results that characterize significant losses in WTP buying and rental values for properties in flood vulnerable locations (A, B, and D) resonate with the findings that property prices tend to be discounted for properties situated in floodplains (Atreya & Ferreira, 2015; Speyrer & Ragas, 1991) and affected by flood inundation (Beltrán et al., 2019). Collectively, our findings on the significant property devaluations that occur in the presence of flood predictions and probabilities provide compelling evidence that there are considerable risks in the provision of such information to the public, perhaps because of their inability to discern underlying flood model uncertainties and interpret probability information (Gourevitch et al., 2023; Rajapaksa et al., 2016; O. Samarasinghe & Sharp, 2010).

Altogether, we consider four models that range from a complex 2D model (LISFLOOD−ROE) to a simple behavioral model (BTM). These models have been set up based on physical site characteristics and recommended flood modeling guidelines. Our flood predictions (Figures 8, 9, 10, 11, 12, 13), thus, represent the best outcomes we can expect from the modeling structures employed. It is beyond the scope of this study to investigate the reasons behind the differences in flood predictions relative to model structure. Instead, what we aim to emphasize here is that various flood maps exist online, specifically for real estate consumers, often informed by different modeling structures (Mehravar et al., 2023; Palm & Bolsen, 2023; Shr & Zipp, 2019). Our results show that: (a) variations in model structure generate differences in flood predictions (Figures 8 and 9); (b) most people appear to make decisions based on extreme flood predictions, perhaps to be risk averse (Belanger & Bourdeau‐Brien, 2017), as evident in the fall in real estate demand for locations A, B, and D, which are erroneously predicted to flood from LISFLOOD−ROE and BTM. Given this, caution is needed when selecting flood models to both inform flood management and communicate flood risk information, as the typical real estate consumer is unlikely to perceive the uncertainty in flood prediction information (Strathie et al., 2015). Essentially, we need to work toward getting flood models “right,” although it is practically impossible to obtain an error‐free model (Jodhani et al., 2023). Hence, much greater care is needed with how flood risk information is communicated to the wider public in order to minimize risks associated with falling real estate demand in response to uncertain flood model predictions. Doyle et al. (2019) argue that when outputs from proprietary systems and analysis platforms on hazard and impact models are presented to decision‐makers without their companion assumptions and underlying uncertainties, it has the potential to compromise their decision‐making capability and limit their usefulness. For instance, there are two distinct aspects of flood prediction—whether an area will flood or not, and the uncertainty in that estimation—that can have different influences on real estate decisions. Our robustness study, however, shows that the provision of flood probabilities replicates the impact on real estate decisions that we observed with the provision of binary flood maps, which classified areas into flood and non‐flood zones, without accompanying probability information. Of concern for the real estate market, this finding suggests that people view flood risk communications through a binary lens, either considering locations to flood or not flood, failing to consider associated probabilities or mis‐interpreting such information to mean that an area will “actually” flood if it appears with an estimated flood risk (even if the risk is low) in these communications. Therefore, an interesting avenue for future work, beyond the presentation of binary flood maps and flood risk probability maps, is to determine how the communication of flood modeling assumptions and uncertainties affects real estate decision‐making of the layperson.

## 6 CONCLUSIONS

We investigate the potential influence of access to multiple sources of flood predictions on residential coastal real estate demand decisions in the United Kingdom by adopting an interdisciplinary approach, involving flood modeling, novel experimental WTP real estate surveys of UK residents in response to hypothetical flood scenarios, statistical modeling, and geospatial analysis. Our findings show that, in the absence of flood prediction information, WTP values are notably higher for beachfront properties, as the majority of people prefer locations with a sea view, than for properties away from the sea. Importantly, the reverse is true when flood prediction information becomes available, despite the uncertainty in these predictions. These findings, which have been validated from our robustness study on real estate decision‐making in response to an actual open‐access flood probability map, suggest that

1. Flood prediction information dominates real estate demand decisions relative to personal preferences (e.g., location aesthetics, convenience, and seclusion) reflecting a shift in real estate demand toward risk averse locations.
2. Flood prediction uncertainty does not factor into real estate demand decision‐making, reflecting a potential inability to perceive flood prediction uncertainty. Although we do not provide explicit information on flood prediction uncertainty (e.g., prediction accuracies and flood event probability) to our survey respondents, the uncertainty in these predictions is evident from the conflicting information in the flood maps provided (Figures 8 and 9), with LISFLOOD−ROE and BTM flood maps showing more extreme flood predictions than those from LISFLOOD−ACC and LISFLOOD−FL. The conflicting information in these maps did not seem to factor into WTP real estate decisions. For instance, if the uncertainty in these flood maps had been perceived (i.e., by recognizing the conflicting information), it is likely that access to these maps would not have had any significant impact on WTP real estate decisions. However, the considerable changes to WTP decisions in response to these maps (Tables 3, 4, 5, 6, 7) indicate, by and large, a failure to detect the uncertainty in flood model predictions. Essentially, we see WTP real estate decisions respond more to extreme predictions.

Flood modelers and managers, therefore, need to be cautious with respect to: (a) how flood predictions are used to inform flood risk management, and (b) how flood prediction information is communicated to the wider public. This requires significant efforts to get flood models “right,” as there are considerable risks of falling real estate demand in response to uncertain flood predictions that are openly available. However, getting flood models “right” is a contentious issue as science is not static and because it means having an error‐free model, which require error‐free input data and discretization. As this is practically impossible, greater efforts are needed to effectively communicate flood information and their uncertainty to the general public.

## CONFLICT OF INTEREST STATEMENT

The authors declare no conflicts of interest.

## ETHICS STATEMENT

Our survey received ethics clearance from Coventry University Research Ethics Committee (ethics project reference: P149798).

## Supporting information



Supporting Information

Supporting Information

Supporting Information

Supporting Information

Supporting Information

Supporting Information
