# Supplementary material for: Decision‐making under flood predictions: A risk perception study of coastal real estate
Source: Risk Anal. 2025 Jan 18;45(7):1899–925. doi: 10.1111/risa.17706 (PMC12396945; doi:10.1111/risa.17706)
Supplement: Supplementary file 4 — Supporting Information [file RISA-45-1899-s005.pdf]

README

This README document describes the contents of the following Stata files:

- **FloodPredictionsAndRealEstateDemand.do** – a Stata do file
- **FloodPredictionsAndRealEstateDemand.dta** – a Stata data file.
- **FloodPredictionsAndRealEstateDemandRobustnessSample.dta** – a Stata data file.

The *Stata do file* provides the commands that allow a user to reproduce the following results in the paper "Decision-making under flood predictions: a risk perception study of coastal real estate":

- **Table 3** Location preference under alternative hypothetical scenarios
- **Table 5** Willingness to buy and rent coastal properties under alternative scenarios
- **Table 6** Paired sample $t - test$ for mean differences in WTP between the baseline and coastal flood scenarios
- **Table 7** Agreeability that flood predictions influenced location preference

This do file also contains detailed descriptions of the variable names, as well as coding and abbreviations used.

The two *Stata data file*s contains: (a) the primary data collected from the novel experimental willingness to pay real estate survey of UK residents associated with the study (FloodPredictionsAndRealEstateDemand.dta) and (b) the additional robustness experiment survey sample (FloodPredictionsAndRealEstateDemandRobustnessSample.dta). These data files contain the responses of participants to hypothetical locations of a coastal property in a quintessential UK coastal town and how they update these decisions under various flood prediction scenarios. Replicators are referred to the associated manuscript, the online survey questionnaire extract document (WTPsurvey.pdf), and the abovementioned Stata do and data files for further information. Additional replication support can be provided upon reasonable request to the corresponding author.

The authors of the manuscript declare that they possess legitimate access and permissions to the data and software used in this manuscript. These include institutional ethics approval to conduct the anonymous online survey and Stata18 software licence.
