# Supplementary material for: Decision‐making under flood predictions: A risk perception study of coastal real estate
Source: Risk Anal. 2025 Jan 18;45(7):1899–925. doi: 10.1111/risa.17706 (PMC12396945; doi:10.1111/risa.17706)
Supplement: Supplementary file 5 — Supporting Information [file RISA-45-1899-s006.pdf]

About your residency

Thank you for continuing. Please note that you cannot change your answers on the previous page. Attempting to return to the previous page will close the survey, and your answers will be lost.

5. Are you a **UK national or have indefinite leave to remain**? Please note that the UK includes England, Northern Ireland, Scotland, and Wales. *

- ◯ Yes
- ◯ No

6. If you selected No, please provide your nationality (i.e. your country of origin and permanent residence): *

7. Please provide the **FIRST PART** of your **CURRENT POSTCODE** (e.g., CV1): *

If you move to the next page, you will not be able to return to this page.

Your willingness to buy and rent a coastal property

Thank you for continuing. Please note that you cannot change your answers on the previous page. Attempting to return to the previous page will close the survey, and your answers will be lost.

The following questions are based on a **hypothetical scenario** that you are either interested in buying or renting a property in a coastal town. Here is an overview map of an undisclosed coastal town showing the number of buildings in the area and their distance from the sea:

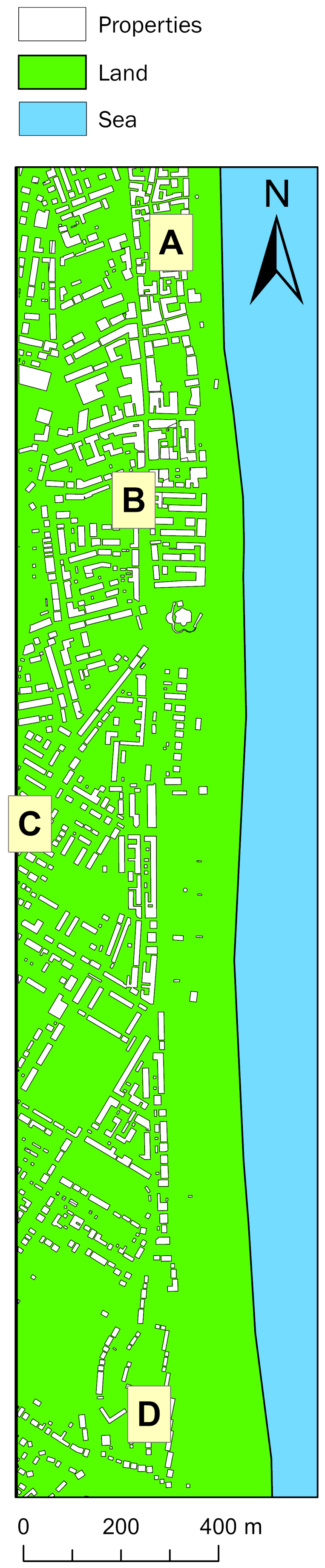

A two-bedroom house in this coastal town has an **average selling price of £275000** and an **average rental price of £975 per month**.

The area has more than **2 miles of mixed shingle and sand beaches**, with wide paved boardwalks.

You can expect to find **all local amenities**, including shopping centres, health care services (e.g., pharmacies, GP), emergency services (e.g., fire), protective services, childcare services, schools, personal and self-care services (e.g., salons), hospitality services (e.g., food outlets), etc.

The area is also located within relatively **close proximity** to a **public university** and **two major international airports**. It is **well-serviced** by public transportation (e.g., buses and trains).

8. How much will you be willing to pay to **BUY** a property at **Location A** in the map above? Note that the average selling price in this coastal town is £275000. *

9. How much will you be willing to pay to **BUY** a property at **Location B** in the map above? Note that the average selling price in this coastal town is £275000. *

10. How much will you be willing to pay to **BUY** a property at **Location C** in the map above? Note that the average selling price in this coastal town is £275000. *

11. How much will you be willing to pay to **BUY** a property at **Location D** in the map above? Note that the average selling price in this coastal town is £275000. *

12. How much will you be willing to pay to **RENT** a property at **Location A** in the map above? Note that the average monthly rent for a property in this coastal town is £975? *

13. How much will you be willing to pay to **RENT** a property at **Location B** in the map above? Note that the average monthly rent for a property in this coastal town is £975? *

14. How much will you be willing to pay to **RENT** a property at **Location C** in the map above? Note that the average monthly rent for a property in this coastal town is £975? *

15. How much will you be willing to pay to **RENT** a property at **Location D** in the map above? Note that the average monthly rent for a property in this coastal town is £975? *

16. Which location on the map would you **most prefer** to live in? *

- ○ Location A
- ○ Location B
- ○ Location C
- ○ Location D

17. What is the **main factor** that influenced your most preferred choice of location? *

If you move to the next page, you will not be able to return to this page.

Your willingness to buy and rent a coastal property (continued)

Thank you for continuing. Please note that you cannot change your answers on the previous page. Attempting to return to the previous page will close the survey, and your answers will be lost.

The map below shows the **long term flood risk** for the same coastal town from tidal surges. The long term flood risk prediction is from a computer model that is used to guide flood management in the UK.

Note: If you are using a phone, you may need to **swipe right** to see the entire map below.

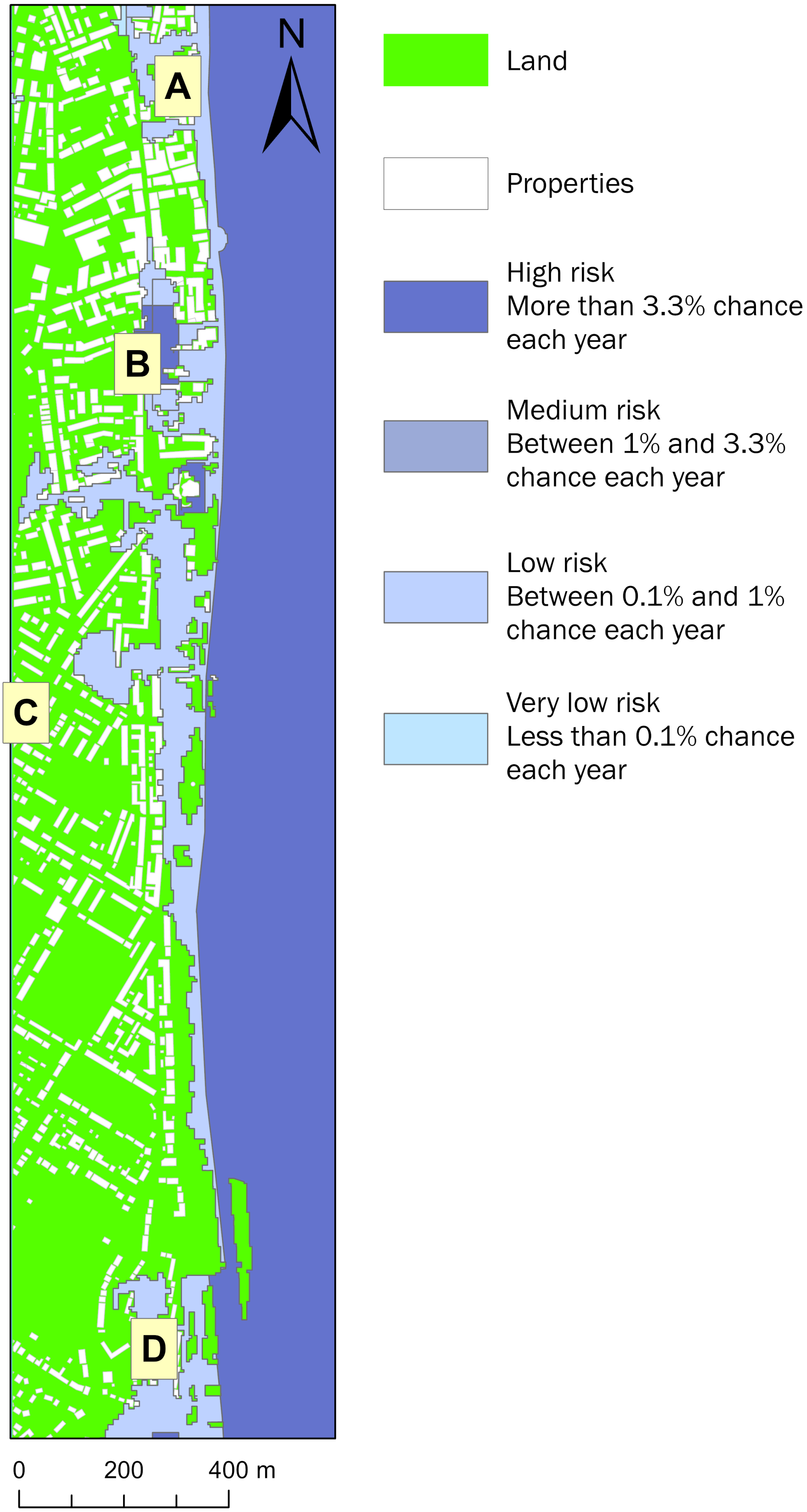

18. Considering the **long term flood risk information**, how much will you now be willing to pay to **BUY** a property in **Location A** in the map above? Note that the average selling price in this coastal town is £275000. *

19. Considering the **long term flood risk information**, how much will you now be willing to pay to **BUY** a property in **Location B** in the map above? Note that the average selling price in this coastal town is £275000. *

20. Considering the **long term flood risk information**, how much will you now be willing to pay to **BUY** a property in **Location C** in the map above? Note that the average selling price in this coastal town is £275000. *

21. Considering the **long term flood risk information**, how much will you now be willing to pay to **BUY** a property in **Location D** in the map above? Note that the average selling price in this coastal town is £275000. *

22. Considering the **long term flood risk information**, how much will you now be willing to pay to **RENT** a property in **Location A** in the map above? Note that the average monthly rent for a property in this location is £975. *

23. Considering the **long term flood risk information**, how much will you now be willing to pay to **RENT** a property in **Location B** in the map above? Note that the average monthly rent for a property in this location is £975. *

24. Considering the **long term flood risk information**, how much will you now be willing to pay to **RENT** a property in **Location C** in the map above? Note that the average monthly rent for a property in this location is £975. *

25. Considering the **long term flood risk information**, how much will you now be willing to pay to **RENT** a property in **Location D** in the map above? Note that the average monthly rent for a property in this location is £975. *

26. Considering the **long term flood risk**, which location on the map will now be your **most preferred** location to live? *

- ◯ Location A
- ◯ Location B
- ◯ Location C
- ◯ Location D

27. To what extent do you agree with the following statement? "The **long term flood risk information** have influenced my choice of location." *

- ◯ Definitely agree
- ◯ Mostly agree
- ◯ Neither agree nor disagree
- ◯ Mostly disagree
- ◯ Definitely disagree

If you move to the next page, you will not be able to return to this page.
